# Micro-aeration for improving anaerobic treatment and biogas production from organic pollutants

**DOI:** 10.1007/s00253-025-13519-w

**Published:** 2025-05-30

**Authors:** Luís Costa, M. Salomé Duarte, Carla Pereira Magalhães, M. Alcina Pereira, Ana Júlia Cavaleiro

**Affiliations:** 1 CEB - Centre of Biological Engineering, University of Minho, Campus de Gualtar, Braga, Portugal; 2 LABBELS – Associate Laboratory, Braga/Guimarães, Portugal

**Keywords:** Anaerobic digestion, Oxygen dosing, Organic pollutants, Lipids, Methane

## Abstract

**Abstract:**

Anaerobic digestion (AD) is a well-established method for waste/wastewater treatment and biogas production, but challenges remain in improving its performance, particularly for toxic/inhibitory organic compounds such as lipids, hydrocarbons, polyphenols, pharmaceuticals, and other pollutants. Micro-aeration, which involves the controlled introduction of small amounts of oxygen, has emerged as a promising strategy to enhance microbial activity, stimulate the degradation of challenging compounds, and improve methane yields. This review addresses the different strategies used for effective oxygen dosing, measurement, and control, while also delving into the bioenergetics of the coexisting anaerobic and micro-aerobic pathways. Studies demonstrating the potential of micro-aeration to enhance the anaerobic treatment of recalcitrant organic pollutants, such as BTEX (benzene, toluene, ethylbenzene, and xylene) and pharmaceutical compounds, are reviewed. Several works use micro-aeration as a pretreatment, while those implementing it directly within bioreactors typically apply it intermittently. Nevertheless, in most cases, the application of micro-aeration is guided by a “trial-and-error” approach, and a systematic understanding of optimal strategies and dosing for different classes of pollutants remains lacking. The review also explores the diverse roles of micro-aeration in the AD of lipids, highlighting key microorganisms and underlying mechanisms that drive these processes, for instance, the role of facultative anaerobes in converting oleate into palmitate and protecting methanogenic communities. Finally, this work highlights future directions and remaining challenges in applying micro-aeration for the anaerobic treatment of organic pollutants.

**Key points:**

- *AD of lipids, hydrocarbons, dyes, pharmaceuticals, and other pollutants is challenging.*
- *Micro-aeration reshapes microbial communities and enhances pollutant degradation.*
- *Effective micro-aeration depends on several factors and is not fully mastered.*

**Graphical abstract:**

**Figure:**
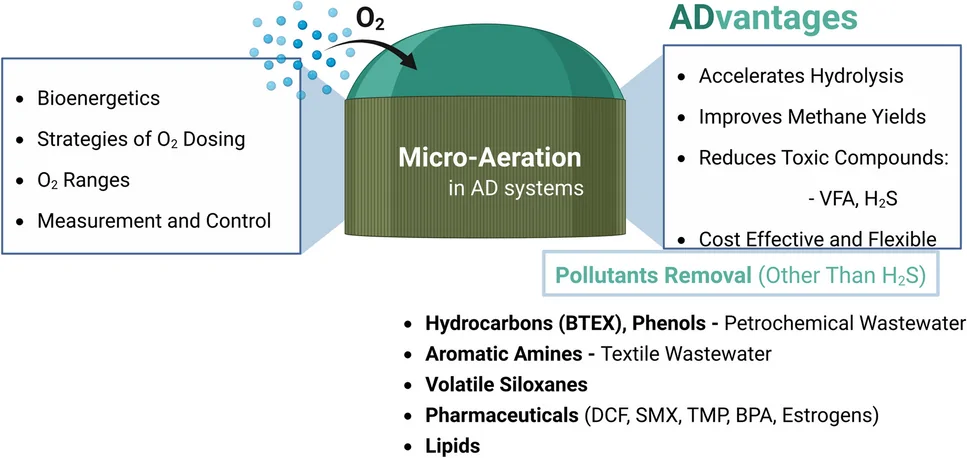

**Supplementary information:**

The online version contains supplementary material available at 10.1007/s00253-025-13519-w.

## Introduction

Anaerobic digestion (AD) is a well-established process for waste/wastewater treatment and renewable energy production, primarily through biogas generation, which can be upgraded to biomethane and used as a sustainable alternative to natural gas. In recent years, global geopolitical tensions and the urgent need to mitigate climate change have driven a shift towards clean energy sources, reducing reliance on fossil fuels (European Commission 2022). This transition is particularly critical in regions heavily dependent on energy imports, such as Europe, where energy security and sustainability are key concerns. The European Commission, for instance, has highlighted biomethane from organic waste as a key element in reducing fossil fuel dependency and decarbonizing the energy sector (European Biogas Association 2024). Additionally, digestate is produced during AD and contributes to nutrient cycling and sustainable agriculture (Briones and Raskin 2003; Chen et al. 2020).

Despite its well-established benefits, AD processes are often constrained by inhibitors, such as volatile fatty acids (VFA) accumulation, which can disrupt microbial activity and compromise overall process stability (Xu et al. 2014; Nguyen et al. 2019). Additionally, the production of hydrogen sulphide (H_2_S) poses a significant challenge, as it is toxic to microorganisms and can destabilize the anaerobic digestion process (Chen et al. 2008). Moreover, the presence of toxic or inhibitory compounds (e.g. hydrocarbons, pharmaceuticals, ammonia, lipids) often compromise the chain of reactions that ultimately lead to methane production. Anaerobic digestion relies on a complex interplay of different microbial groups that sequentially break down organic matter into simpler compounds. This process involves four main stages: hydrolysis, acidogenesis, acetogenesis, and methanogenesis. Each stage depends on the previous one, and the inhibition of any microbial group can disrupt the entire process, reducing methane yields and overall system efficiency (Chen et al. 2020).

Studies have explored innovative strategies to optimize AD performance, with micro-aeration emerging as a promising approach. Micro-aeration involves the controlled introduction of small amounts of oxygen into an anaerobic environment. This approach can enhance microbial activity, boost hydrolysis, improve methane yields, promote the degradation of VFA and the removal of toxic compounds, and reduce H_2_S accumulation (Cavaleiro et al. 2016; Nguyen and Khanal 2018; Duarte et al. 2020). Therefore, micro-aeration not only addresses operational challenges but also holds potential for improving the economic and environmental viability of AD systems (Fu et al. 2018b; Song et al. 2021).

Despite its advantages, micro-aeration requires careful implementation, as oxygen exposure can negatively affect strict anaerobes, such as methanogens and sulphate-reducing bacteria (SRB), which thrive in low redox environments (Jasso-Chávez et al. 2015). SRB generate hydrogen sulphide, which is toxic for methanogens, and they also outcompete methanogens for key substrates such as acetate and H_2_. Therefore, micro-aeration might be exploited to enhance methanogenic processes by limiting SRB activity, provided methanogen inhibition by O₂ can be minimized, for example, in a two-phase system. Maintaining a balance that supports the synergistic interactions between the different microbial key players involved in efficient organic matter conversion to methane is thus required (McInerney et al. 2009; Sieber et al. 2012).

This review provides a practical perspective on micro-aeration in AD, examining its implementation, operational challenges, and benefits in optimizing waste-to-energy processes, with a special focus on AD of toxic/recalcitrant compounds, since this topic has received less attention compared to other AD applications (e.g. improving hydrolysis or removing H_2_S). Various terms are presented in the literature to describe the introduction of oxygen into the AD process. Some authors distinguish between the terms “micro-aeration” and “micro-oxygenation”, where the former refers to the dosing of air into the anaerobic reactor and the latter refers to the use of pure oxygen. In this paper, the term “micro-aeration” is generally used to describe both approaches of introducing small quantities of oxygen in AD processes.

## Bioenergetics in anaerobic and micro-aerobic environments

Micro-aeration in AD creates a hybrid environment where both anaerobic and aerobic conditions coexist, supporting beneficial niches for both strict and facultative anaerobes, as well as to micro-aerobes (Ortiz-Ardila et al. 2021). Under strict anaerobic conditions, organic matter is only partially transformed into energy-rich molecules due to the lack of an efficient electron transport chain, resulting in lower energy yields from organic degradation reactions. In fact, in these environments, macromolecules (proteins, sugars, and lipids) undergo hydrolysis and fermentation, producing intermediary compounds such as alcohols and VFA. These intermediates are further converted into acetate and hydrogen/formate. However, the accumulation of these products imposes thermodynamic constraints. This is evident in the positive Gibbs free energy change associated with these reactions—e.g. for the oxidation of ethanol, butyrate, propionate, and oleate to acetate and hydrogen, Δ*G*^0'^ is + 9.6, + 48.3, + 24.2, and + 325.7 kJ reaction^−1^, respectively, under standard conditions and pH 7 (Thauer et al. 1977; Cavaleiro et al. 2016; Nguyen and Khanal 2018). Such thermodynamic limitations are generally overcome through syntrophic interactions, wherein a microbial partner, commonly a hydrogenotrophic methanogen, consumes the hydrogen or formate produced. This consumption shifts the reaction equilibrium, rendering exergonic (Δ*G*^0'^ < 0 kJ reaction^−1^) previously endergonic reactions and thereby enabling continued degradation of the macromolecules and their intermediates (Table 1). This is also the case for other possible toxic organic compounds, e.g. benzene and toluene (Zengler et al. 1999; Vogt et al. 2011). Nevertheless, the reduced energetic gain from the overall methanogenic reactions (Table 1) open spaces for a positive effect of micro-aeration in AD.

**Table 1:** Energetics of the conversion reactions for major classes of biological macromolecules under anaerobic, aerobic, and micro-aerobic conditions

Model substrate		Reaction under aerobic/micro-aerobic conditions	Δ*G*^0′a^ kJ react.^−1^	Δ*G*′^b^ kJ react.^−1^
Proteins	Creatine	C_4_H_9_N_3_O_2_ + 3 O_2_ → 4 CO_2_ + 3 NH_3_	− 1392.9	− 1299.3
Carbohydrates	Sucrose	C_12_H_22_O_11_ + 12 O_2_ → 12 CO_2_ + 11 H_2_O	− 5789.4	− 5415.2
Lipids	Oleate^c^	C_18_H_33_O_2_^−^ + H^+^ + 25.5 O_2_ → 18 CO_2_ + 17 H_2_O	− 10,916.1	− 10,120.9
Hydrocarbons	Benzene^c^	C_6_H_6_ + 7.5 O_2_ → 6 CO_2_ + 3 H_2_O	− 3202.2	− 2941.6
Hydrocarbons	Toluene^c^	C_7_H_8_ + 9 O_2_ → 7 CO_2_ + 4 H_2_O	− 3823.5	− 3509.0
Intermediary products				
Amino acids	Alanine	C_3_H_7_NO_2_ + 3 O_2_ → 3 CO_2_ + NH_3_ + 2 H_2_O	− 1312.5	− 1210.7
Amino acids	Glycine	C_2_H_5_NO_2_ + 1.5 O_2_ → 2 CO_2_ + NH_3_ + H_2_O	− 681.7	− 631.5
Amino acids	Leucine	C_6_H_13_NO_2_ + 7.5 O_2_ → 6 CO_2_ + NH_3_ + 5 H_2_O	− 3234.7	− 3181.9
Monosaccharides	Glucose	C_6_H_12_O_6_ + 6 O_2_ → 6 CO_2_ + 6 H_2_O	− 2872.0	− 2658.2
Alcohols	Ethanol	C_2_H_5_OH + 3 O_2_ → 2 CO_2_ + 3 H_2_O	− 1318.5	− 1221.5
VFA	Butyrate	C_4_H_7_O_2_^−^ + H^+^ + 5 O_2_ → 4 CO_2_ + 4 H_2_O	− 2133.7	− 1964.0
VFA	Propionate	C_3_H_5_O_2_^−^ + H^+^ + 3.5 O_2_ → 3 CO_2_ + 3 H_2_O	− 1493.7	− 1376.3
VFA	Acetate	C_2_H_3_O_2_^−^ + H^+^ + 2 O_2_ → 2 CO_2_ + 2 H_2_O	− 853.8	− 788.0
−	H_2_	H_2_ + 0.5 O_2_ → H_2_O	− 237.2	− 221.6
Model substrate		Reaction under anaerobic (methanogenic) conditions	Δ***G***^0′a^ kJ react.^−1^	−
Proteins	Creatine	C_4_H_9_N_3_O_2_ + 3 H_2_O → 1.5 CH_4_ + 2.5 CO_2_ + 3 NH_3_	− 165.9	−
Carbohydrates	Sucrose	C_12_H_22_O_11_ + H_2_O → 6 CH_4_ + 6 CO_2_	− 881.6	−
Lipids	Oleate^c^	C_18_H_33_O_2_ + H^+^ + 8.5 H_2_O → 12.75 CH_4_ + 5.25 CO_2_	− 527.0	−
Hydrocarbons	Benzene^c^	C_6_H_6_ + 4.5 H_2_O → 3.75 CH_4_ + 2.25 CO_2_	− 134.8	−
Hydrocarbons	Toluene^c^	C_7_H_8_ + 5 H_2_O → 4.5 CH_4_ + 2.5 CO_2_	− 142.6	−
Intermediary products				
Amino acids	Alanine	C_3_H_7_NO_2_ + H_2_O → 1.5 CH_4_ + 1.5 CO_2_ + NH_3_	− 85.5	−
Amino acids	Glycine	C_2_H_5_NO_2_ + 0.5 H_2_O → 0.75 CH_4_ + 1.25 CO_2_ + NH_3_	− 68.2	−
Amino acids	Leucine	C_6_H_13_NO_2_ + 2.5 H_2_O → 3.75 CH_4_ + 2.25 CO_2_ + NH_3_	− 167.4	−
Monosaccharides	Glucose	C_6_H_12_O_6_ → 3 CH_4_ + 3 CO_2_	− 418.1	−
Alcohols	Ethanol	C_2_H_5_OH → 1.5 CH_4_ + 0.5 CO_2_	− 91.6	−
VFA	Butyrate	C_4_H_7_O_2_^−^ + H^+^ + H_2_O → 2.5 CH_4_ + 1.5 CO_2_	− 88.7	−
VFA	Propionate	C_3_H_5_O_2_^−^ + H^+^ + 0.5 H_2_O → 1.75 CH_4_ + 1.25 CO_2_	− 62.2	−
VFA	Acetate	C_2_H_3_O_2_^−^ + H^+^ → CO_4_ + CO_2_	− 35.8	−
−	H_2_	H_2_ + 0.25 CO_2_ → 0.25 CH_4_ + 0.5 H_2_O	− 32.7	−

^a^Gibbs free energy change (Δ*G*^*0′*^) was calculated under standard conditions (25 °C, solute concentrations of 1 mol L^−1^, gas partial pressure of 1 atm, and H^+^ concentration at pH 7). Data obtained or calculated from Thauer et al. (1977)

^b^Δ*G*′ was calculated with solute concentrations of 1 mol L^−1^, oxygen concentration of 3.41 µmol L^−1^ (equivalent to 1% air-saturated water at sea level (1 atm) and 4 °C), gas partial pressure of 1 atm, pH 7, at 25 °C

^c^Representative of toxic compounds

Aerobic conditions promote the rapid consumption of organic compounds and microbial growth, driven by a highly active metabolism (Nguyen and Khanal 2018). The associated biochemical reactions are highly exergonic, as evidenced by the negative values of Δ*G*^0'^ for the aerobic degradation of key biological macromolecules (proteins, sugars, and lipids), as well as some typical AD intermediaries (amino acids, monosaccharides, alcohols, VFA) and potentially toxic organic compounds (benzene and toluene) (Table 1). These values are often more than one order of magnitude higher than those observed under anaerobic methanogenic conditions (Table 1), highlighting the energetic advantage of aerobic metabolism.

**Fig. 1:**
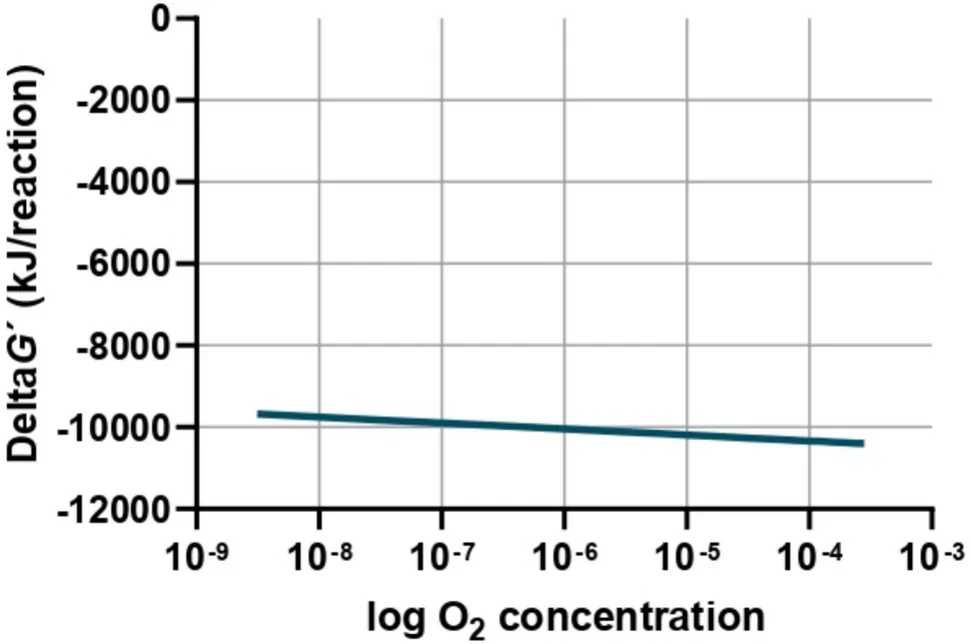
Gibbs free energy change with oxygen concentration, for the reaction of aerobic oleate oxidation under non-standard conditions (at 25 °C, oleate concentration of 1 mol L^−1^, gas (other than O_2_) partial pressure of 1 atm, pH 7)

The Gibbs free energy change under micro-aerobic conditions (1% air-saturated water at sea level (1 atm) and 4 °C) was also calculated for the same organic compounds (Δ*G*′, Table 1). Under these conditions, the reactions are only slightly less favourable compared to fully aerobic conditions, and the effect of decreasing O_2_ concentrations in Δ*G*′ is relatively small, as illustrated in Fig. 1, using the reaction of aerobic oleate oxidation as an example. This indicates that such reactions remain significantly more favourable than under anaerobic conditions. These findings suggest that even small amounts of oxygen can enhance the efficiency of the biochemical processes and stimulate specific microbial groups that thrive under low oxygen conditions, by enhancing their metabolic activity. For example, Nguyen et al. (2019) pointed out that facultative bacteria can shift from anaerobic fermentation to the more energetically favourable aerobic respiration upon intermittent O_2_ dosing. This is possibly related with the ability of some prokaryotes to grow aerobically at nanomolar O_2_ concentrations (Wu et al. 2021).

Consequently, the limited oxygen availability under micro-aerobic conditions may promote the occurrence of partial biochemical pathways (e.g. hydrolysis), but, in some cases, it may also allow complete reactions to occur (e.g. aerobic oxidation of VFA). However, in these cases, oxygen acts as limiting reagent, thereby constraining the overall extension of the reaction. This is particularly relevant for avoiding substrate and energy diversion away from methane production, while simultaneously reducing the accumulation of toxic or inhibitory compounds. Therefore, integrating controlled and balanced aerobic oxidation by heterotrophs with anaerobic degradation pathways and methanogenesis emerges as a promising strategy to enhance energy transformations while maintaining stability within AD processes. The interplay between micro-aerobic bacteria and strict anaerobes, including syntrophic bacteria and methanogenic archaea, is thus highly relevant. This topic has been thoroughly reviewed by Nguyen and Khanal (2018), and therefore it will not be addressed in detail in this review.

## Micro-aeration strategies

### Different ways to achieve micro-aeration

**Fig. 2:**
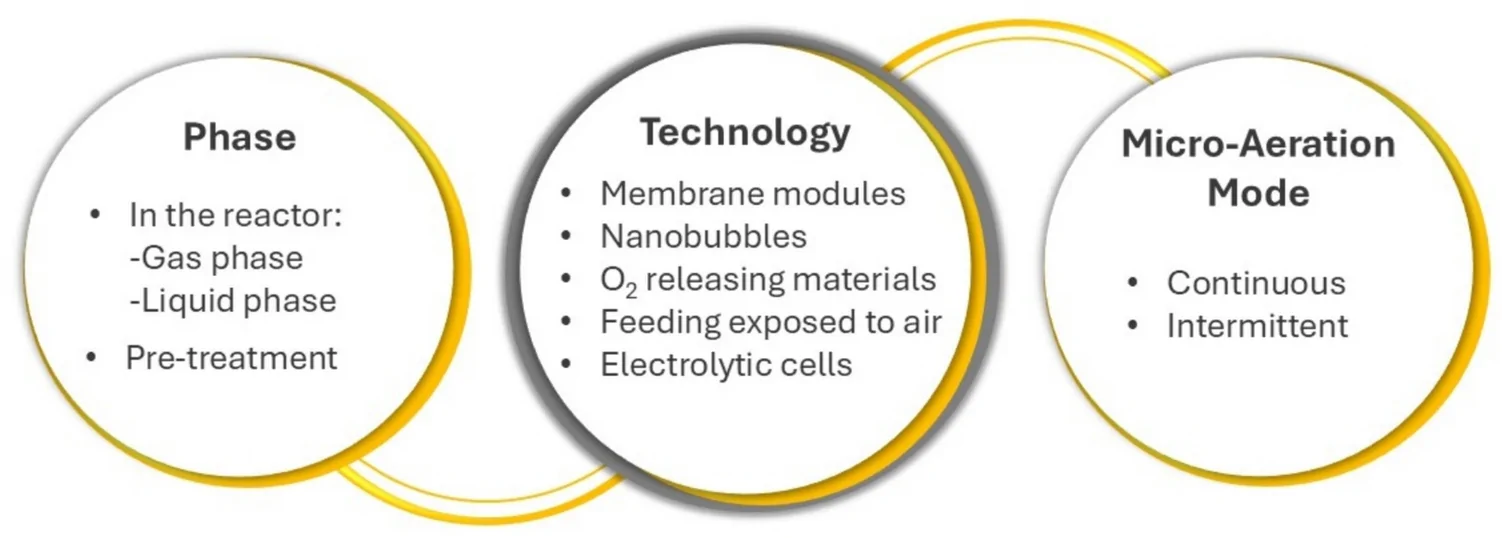
Overview of micro-aeration application points, techniques, and application modes in AD processes

Efficient oxygen dosing methods are crucial for optimizing micro-aeration in AD; however, detailed descriptions of these methods remain limited in the literature. Micro-aeration can be applied directly within a single anaerobic bioreactor—where all microbiological phases occur simultaneously—or as a pretreatment step (Fig. 2). In the latter case, micro-aeration is typically used to promote hydrolysis and acidification of the substrate in a first stage, followed by a second anaerobic stage. The introduction of small amounts of oxygen is commonly done either into the gas phase, in the headspace of the bioreactors, or directly into the liquid phase (Fig. 2). Each approach presents distinct advantages and challenges.

Oxygen introduction into the gas phase typically requires less energy. This approach is commonly applied to remove H_2_S, since it was identified as the most effective method (Kobayashi et al. 2012). This efficiency arises because oxygen is utilized by sulphur-oxidizing bacteria (SOB), which oxidize H_2_S to elemental sulphur (S⁰) or sulphate (SO_4_^2−^). These bacteria thrive in the upper region of the digestor, where both oxygen and H_2_S are present. As a result, the volume of pure oxygen or air required to treat a given amount of H_2_S is reduced. Consequently, this minimizes nitrogen contamination in the biogas, enhancing its quality (Krayzelova et al. 2015).

For other applications, micro-aeration in the gas phase is implemented under batch/fed-batch conditions, often at mild overpressure levels, and sufficient time should be provided for the oxygen in the headspace to equilibrate and diffuse across the liquid medium. These conditions minimize the risk of localized high O_2_ concentrations, thereby helping to prevent acute toxicity to strict anaerobes (Di Costanzo et al. 2024). In these systems, the gradual consumption of oxygen by microorganisms promotes continued diffusion and a steady transfer of oxygen from the gas phase to the liquid. On the other hand, injecting oxygen directly into the liquid phase can enhance the transfer rate and promote higher microbial activity by oxygen-tolerant organisms but requires more energy and advanced equipment to ensure efficient gas dispersion (Buakaew and Ratanatamskul 2024; Hou et al. 2024). The introduction of pure oxygen or air bubbles into an anaerobic reactor may irreversibly inhibit methanogens, if freely dispersed in the liquid phase, due to their strictly anaerobic nature (Botheju et al. 2010; Botheju 2011). However, recent studies have shown that some methanogens possess mechanisms to mitigate oxidative stress, including enzymes such as catalase (KAT) and superoxide dismutase (SOD), which neutralize reactive oxygen species (ROS) (Pedone et al. 2004). Oxygen tolerance varies significantly among different methanogenic groups. For example, Lyu and Lu (2018) categorized methanogens into two groups based on their genetic adaptations: Class I (including Methanobacteriales, Methanococcales, and Methanopyrales) and Class II (comprising Methanocellales, Methanomicrobiales, and Methanosarcinales). Class II methanogens exhibit greater adaptability to oxidative environments, partly because they use enzymes that minimize ROS generation or improve ROS removal (Lyu and Lu 2018). Additionally, in anaerobic digesters, methanogens are generally growing in aggregates (granules) or as biofilms, thus benefiting from the protection provided by outer layers of oxygen-tolerant bacteria, which consume oxygen before it reaches the inner layers (Magdalena et al. 2022; Buakaew and Ratanatamskul 2024). The selection of the method for gas dispersion is crucial, as micro-aeration requires effective gas transfer due to oxygen’s low solubility in water and its limited gas-to-liquid phase transfer efficiency. To address the oxygen diffusion limitation in the gas–liquid interface, several strategies have been applied (Fig. 2), namely the use of oxygen-transferring membranes for controlled oxygen dosing (Pokorna-Krayzelova et al. 2018; Fu et al. 2023). Membrane-aerated reactors use membranes of different materials, such as silicone tubes or polypropylene membranes, to deliver oxygen efficiently with more uniform mass transfer. Through the membrane module, pressurized gas diffuses into the solution without forming bubbles, achieving an approximately 100% oxygen transfer rate (OTR) (Perez-Calleja et al. 2017; Zhang et al. 2022), with low energy consumption thus overcoming the problems faced in wastewater treatment (Syron et al. 2015). The membranes can also promote microbial colonization on their rough surfaces, leading to biofilm development. This may be beneficial for biomass retention, but it can also contribute to membrane fouling over time, thus representing both an advantage and a potential limitation. Recent studies have explored membrane-based micro-aeration as an effective method for enhancing the degradation of refractory organic matter in anaerobic systems. For instance, Zhang et al. (2022) demonstrated that membrane-based micro-aeration (0.5–5.0 mL min^−1^ air supply) significantly improved the degradation of 2,4-dinitrophenol in a hydrolysis acidification reactor, achieving 2 to 3% higher degradation rates compared to traditional bubble aeration methods. Bubble-free membrane micro-aeration was also successfully applied by Wei et al. (2024) in an anaerobic membrane bioreactor (AnMBR), to increase the hydrolysis and acidification of insoluble starch and soluble peptone, which lead to 66–145% increase in methane production. Additionally, this approach also raised the critical flux of the microfiltration membrane by driving large floc formation, improving sludge filterability.

Another innovative approach being explored in combination with AD is the use of nanobubble (NB) technology (Fig. 2), which generates bubbles with diameters ranging from 50 to 200 nm (Atkinson et al. 2019; Chuenchart et al. 2021). The gaseous spherical bubbles (N_2_, CO_2_, H_2_, O_2_, air) are usually produced by hydrodynamic cavitation. They have higher interfacial area per volume of gas and longer lifetimes in water (2 weeks or more) compared to larger bubbles, resulting in more efficient gas partitioning into water (Atkinson et al. 2019; Hou et al. 2024). This technology has been shown to be promising for increasing biodegradability rates, methane and hydrogen production, and hydrolytic enzyme activity during pretreatment tests with several substrates, such as cellulose (Wang et al. 2020a, 2020b), corn straw (Wang et al. 2020c), and kitchen waste (Hou et al. 2021).

Oxygen-releasing materials (Fig. 2), such as granular calcium peroxide, have also been applied to create micro-aeration conditions in AD, leading to increased methane yields (Fu et al. 2018a), though further research is needed to understand their effects on microbial physiology and long-term AD performance.

Electrolytic aeration (Fig. 2) has also been proposed as a cutting-edge solution for in situ micro-aeration (Fu et al. 2023). This technique involves producing oxygen and hydrogen through electrolytic aeration, with oxygen used to create the micro-aerobic environment and hydrogen serving as a substrate for methane synthesis (Tartakovsky et al. 2011; Nguyen and Khanal 2018). Adjusting the electric current allows the control of aeration levels. For instance, applying a voltage of 2.8–3.5 V in a sludge bed reactor resulted in increased hydrolysis, higher net methane production (10–25%), and improved reactor stability compared to a conventional reactor, as well as enhanced combustion capacities of the biogas, due to the release of a small portion of the H_2_ into the headspace (Tartakovsky et al. 2011).

By using feeding tanks that are not completely sealed (Fig. 2), small oxygen amounts enter the reactor naturally, which can have a very significant impact on AD. Although this system does not allow full control of the oxygen added, it may be closer to what happens in some full-scale AD plants, where strict anaerobic conditions may not consistently be maintained and thus vestigial oxygen may be found. For example, in traditional livestock farms or in the anaerobic digestion of sewage sludge, feedstocks are typically stored in tanks that—although generally covered and non-aerated—are not fully anaerobic. These tanks often maintain anoxic or facultative conditions, with only minimal levels of oxygen present. To simulate this entrance of vestigial oxygen amounts through the feeding, Duarte et al. (2018) operated bioreactors with feeding tanks open to the air (opening area per volume = 1.9–2.5 m^−1^) and found that this approach facilitated the conversion of toxic long-chain fatty acids to methane, when compared to strict anaerobic feeding. The oxidation–reduction potential (ORP) in these microaerophilic feeding tanks was approximately − 100 mV, contrasting with the stricter anaerobic feeding, which exhibited ORP values around − 250 mV. This approach led to an ORP increase from approximately − 350 mV (strict anaerobic) to − 250 mV (microaerophilic), indicating the presence of small oxygen amounts in the system.

Therefore, multiple oxygen dosing strategies show potential for enhancing AD processes, but further research is needed to optimize these methods for industrial-scale application and understand their long-term impacts on AD performance.

### Oxygen levels in the system

Oxygen addition is generally performed by injecting air or pure oxygen (Fig. 2). Air is more commonly used from a cost perspective, as pure oxygen typically incurs additional expenses. Nguyen and Khanal (2018) propose that air is sufficient if the primary purpose is to enhance hydrolysis and VFA production. However, the use of air can lead to undesirable dilution of biogas due to the presence of nitrogen. This issue is particularly challenging when biogas with a low methane content (approximately 50%) is produced (Krayzelova et al. 2015). Consequently, when the objective is to generate high-quality biogas, such as renewable natural gas, pure oxygen may be the preferred option to avoid nitrogen dilution associated with air injection. Additionally, air injection generally necessitates higher dosages due to the lower oxygen content (~ 21%) in comparison with the use of pure oxygen (100%).

Precise management of air or pure oxygen dosage is essential. Facultative anaerobes rely on DO to secrete extracellular hydrolases, making oxygen a limiting factor for their growth. However, high oxygen doses not only inhibit methanogenic microorganisms but also pose an explosion risk, as escaping air or oxygen can concentrate in biogas (Krayzelova et al. 2015; Chen et al. 2020). The explosion range of methane lies between 4 and 17% by volume in air (International Social Security Association 2019). Additionally, excessive oxygen can promote aerobic metabolism of intermediate organic compounds by facultative anaerobes, reducing the availability of substrates for methanogenesis and leading to reduced methane yields, which is undesirable.

Higher concentrations of oxygen around 1.0 mg L^−1^ have been applied in micro-aerobic hydrolysis process to intensify sludge reduction (Niu et al. 2016; Xu et al. 2021) which may be applied as a pretreatment prior to AD process. Micro-aeration applying lower concentrations of oxygen, such as 0.1 mg L^−1^, has been directly applied in AD systems, as shown in Table 2. This range of concentrations has been shown to establish micro-aerobic conditions suitable for the growth of microaerophilic and facultative microorganisms (Wu et al. 2015; Xu et al. 2021). Nguyen and Khanal (2018) reviewed typical oxygen loads (expressed as the volume of O_2_ per unit volume of reactor and time) ranging from 0.005 to 5 L L^−1^ day^−1^. However, these dosages, or the intensity of micro-aeration, can vary depending on several factors, such as the specific objective of the process, the source of the inoculum, the reactor configuration, the substrate type, and the organic loading rate applied (Nguyen and Khanal 2018).

**Table 2:** Summary of studies that applied micro-aeration for anaerobic removal of pollutants, including key parameters such as substrate, reactor configuration, air/oxygen dosage, and observed effects

Substrate	Reactor type	Air/pure O_2_	Pretreatment (Pt) Direct (D)	Aeration^a^	Effect	Reference
Olive mill wastewater	Glass flask (batch)	Air	Pt—continuous (5 or 7 days)	936 L L^−1^ d^−1^	Decrease of phenols (78–90%) Decrease total COD (65%)	González-González and Cuadros (2015)
Synthetic BTEX-contaminated water	UASB	Air	D—intermittent (through feeding line)	0.07, 0.10, and 0.30 L L^−1^ d^−1^	Removal of BTEX (> 83%) Without volatilization	Siqueira et al. ((2018)
Petrochemical wastewater	Digestion reactor	Air	Pt—intermittent (ORP-based)	0.04–0.06 mg L^−1^	Reduced H_2_S Removal of BTEX	Wu et al. (2015)
Petrochemical wastewater	Full scale	Air	Pt—intermittent (ORP-based)	0.08–0.10 mg L^−1^	Increased COD removal (55%) Increased removal of all micropollutants Increased acidogenesis	Wu et al. (2018)
Anionic surfactants	AnMBR	Air	D—intermittent (ORP-based)	NM	Removal of surfactant (80%) No foam No VFA accumulation	Cheng et al. (2018)
2-Butenal manufacture wastewater	EGSB	Air	D—continuous	0.04–0.10 mg L^−1^	COD removal (24%) Increased acidification (21%)	Song et al. (2019)
Textile wastewater (azo dye Direct Black 22)	UASB	Air	D—intermittent (DO monitored daily)	0.04 ± 0.01 mg L^−1^	No difference in dye removal Increased aromatic amines removal Decreased toxicity	Carvalho et al. (2020)
Synthetic pharmaceutical wastewater	UASB	Air	D—intermittent (through feeding line)	0.08 L L^−1^ d^−1^	Increased removal of micropollutants (> 50%) Community not changed	Buarque et al. (2019)
Domestic with pharmaceutical wastewater	Anaerobic baffled biofilm-membrane bioreactor (AnBB-MBR)	Air	D—intermittent (DO monitored)	0.06–0.10 mg L^−1^	Enhanced adsorption and biodegradation of pharmaceuticals Ciprofloxacin (78%) Sulfamethoxazole (89%) Diclofenac (41%)	Buakaew and Ratanatamskul (2024)
Fresh leachate	Sequential reactors (anoxic/micro-aerobic/oxic)	Air	D—intermittent (relay-controlled DO)	< 0.15 mg L^−1^	Increased acetogenesis Improved denitrification Antibiotics removal (50% efficiency)	Wei et al. (2021)
Synthetic wastewater with 2,4-dinitrophenol	Membrane-based bubbleless micro-aeration hydrolysis acidification (MBL-MHA)	Air	D—continuous (through membrane)	0.06–0.60 L L^−1^ d^−1^	Degradation of 2,4-dinitrophenol achieving 2 to 3% higher degradation rates compared to bubble aeration method	Zhang et al. (2022)
WAS with siloxanes	Bach assays	O_2_	D—intermittent (time-based)	1% or 3% (v/v)	Enhanced methane production	Ortiz-Ardila et al. (2024)

*BTEX*; benzene, toluene, ethylbenzene, and xylene, *WAS*; waste activated sludge; *MBL-MHA*; membrane-based bubbleless micro-aeration coupled with hydrolysis acidification, *MABR*; membrane-aerated biofilm reactor, *UASB*; upflow anaerobic sludge blanket, *AnMBR*; anaerobic membrane bioreactor, *EGSB*; expanded granular sludge bed, *NM*; not mentioned, *COD*; chemical oxygen demand, *VFA*; volatile fatty acids, *DO*; dissolved oxygen, *OTR*; oxygen transfer rate

^a^Depending on the information available, aeration is expressed either as the aeration rate (volume of O_2_ per unit of reactor working volume and time), or as the target dissolved oxygen (DO) concentration (mass of O_2_ per unit of reactor working volume) maintained throughout the experiment

Defining the best micro-aeration intensity is key. For instance, Zhu et al. (2009) studied the effects of different micro-aeration ranges and found that the lowest tested intensity (i.e. aeration for 5 min every 24 h at an air flow rate of 700 mL min^−1^, which corresponds to 2.5 L L^−1^ day^−1^) led to a sharp pH decrease and lactic acid accumulation, as well as lower hydrolysis efficiency of lignocellulosic compounds and reactor instability. Since these effects were observed under the lowest micro-aeration condition tested, but not under higher micro-aeration regimes, the authors recommend that insufficient micro-aeration should be avoided during AD of solid wastes (Zhu et al. 2009). Another example is when micro-aeration is used to eliminate H_2_S in bioreactors treating sulphur-rich wastewater, with low chemical oxygen demand (COD) to S ratios, such as those from the brewery, sugar, or paper industries. Due to their low COD/S ratios, these processes have been shown to need more oxygen per unit of biogas for H_2_S removal than bioreactors treating sewage sludge, agricultural residues, or manure (Krayzelova et al. 2015).

Numerous studies have investigated the micro-aeration process, evaluating different oxygen dosages (Table 2, Tables S1 and S2). These studies demonstrate that various substrates can be successfully treated through micro-aeration, either as a pretreatment or by direct O_2_ administration in the reactors.

The solubility of oxygen in water is inversely proportional to temperature. In thermophilic systems, the lower solubility of oxygen at higher temperatures implies that less oxygen can be dissolved in the liquid phase for a given partial pressure of oxygen. Nevertheless, the diffusion rate of oxygen increases with temperature, while the liquid viscosity and surface tension decrease, which can offset the reduction in OTR due to lower solubility, especially at higher temperatures. Reports of micro-aeration in thermophilic anaerobic bioreactors are scarce. Some studies show that thermophilic micro-aerobic conditions can significantly accelerate hydrolysis and increase methane yield. For example, Fu et al. (2015a) reported that a thermophilic micro-aerobic pretreatment of corn straw at 55 °C, with an oxygen load of 5 mL g^−1^ volatile solids (VS) (based on the VS of the residual substrate), resulted in a 17% increase in methane yield and a 11% increase in volatile solid (VS) removal efficiency compared to untreated samples. In addition, structural characterization analyses (FT-IR and XRD) indicated improved hydrolysis efficiency, as the cellulosic structures and pores of corn straw were partially disrupted and a decrease in the crystallinity index was observed. The authors suggest that these structural changes during the thermophilic micro-aerobic pretreatment process are likely responsible for the improved methane yields observed (Fu et al. 2015a). In a subsequent study, the same authors applied a second thermophilic micro-aerobic pretreatment (10 mL g^−1^ VS) when an obvious decrease in daily methane yield was observed. The results showed that the methane production was 28% and 11% higher than that of the untreated and once thermophilic micro-aerobic pretreated samples, respectively. Similarly, the cumulative methane yield increased by 29% and 17% compared to the untreated and once-pretreated samples, respectively (Fu et al. 2015b).

### Measurement and control

Monitoring and controlling micro-aeration conditions require specific oxygen measurement methods, which are crucial for experimental monitoring (Charles et al. 2009; Beyene et al. 2014; Magdalena et al. 2022). The direct methods commonly employ electrochemical and optical sensors, each offering distinct advantages and limitations.

Electrochemical sensors operate by allowing oxygen to diffuse through a permeable membrane into the sensor, where a reduction reaction generates an electrical signal. This signal is analyzed to determine oxygen concentration (Wei et al. 2019). These sensors can measure oxygen in the liquid phase, both directly in the reactor or in samples collected from the reactor (although, in this case, the measurement should be made as fast as possible, to avoid changes). Although highly sensitive, they consume oxygen during the measurement process, which may interfere with analyses at trace levels (Magdalena et al. 2022).

In contrast, optical sensors measure oxygen concentration through fluorescence quenching, enabling measurements in both liquid and gas phases. Oxygen molecules absorb the optical signal emitted by the sensor, thereby preventing fluorescence (Jiang et al. 2017). Optical sensors are particularly advantageous due to their small size, extremely low oxygen detection limits (3.2 × 10^−3^ mg L^−1^), and suitability for non-invasive applications, which minimize contamination risks. These features make them ideal for experiments requiring high sensitivity at trace oxygen levels (Lehner et al. 2014; Magdalena et al. 2022).

Alternatively, some researchers prefer an indirect method, such as the measurement of oxidation–reduction potential (ORP). ORP reflects a compound’s tendency to gain or lose electrons and is expressed in millivolts (mV). This method is advantageous due to its high sensitivity, capable of detecting minimal changes in oxygen concentrations, even at trace levels (Liu et al. 2013; Nguyen and Khanal 2018; Magdalena et al. 2022). Studies show that ORP values correlate with oxygen concentration: for example, − 50 mV corresponds to dissolved oxygen concentrations of 0.1 mg L^−1^ (3 µmol L^−1^) (Liu et al. 2013; Nguyen and Khanal 2018). Thus, typical ORP values for micro-aerobic conditions range from 0 to − 300 mV, whereas values below −300 mV indicate anaerobic conditions and values above 0 mV are associated with aerobic environments (Khanal and Huang 2006; Krayzelova et al. 2015). While membrane and optical sensors are often designed for low-strength wastewater, ORP sensors are particularly practical for high-strength wastewater or semi-solid substrates, where their robustness and ability to handle complex matrices are advantageous. ORP sensors are also useful for monitoring dynamic changes in anaerobic systems (Nguyen and Khanal 2018; Chen et al. 2020). However, they are influenced by environmental parameters such as pH, temperature, and ionic strength. For instance, in systems like anaerobic reactors with microbiomes, changes in substrate composition and microbial activity may affect the accuracy of ORP as monitoring and control parameter (Magdalena et al. 2022). Nonetheless, ORP has been widely applied to control micro-aeration in anaerobic processes, such as wastewater treatment and digestion of lignocellulosic substrates (Zitomer and Shrout 1998; Tsapekos et al. 2017).

## Applications of micro-aeration

A significant proportion of the published works uses micro-aeration to enhance hydrolysis, as shown in Table S1, frequently with the ultimate goal of improving the methane yield. This approach has been successfully tested with several substrates, such as waste activated sludge (WAS) (Hasegawa et al. 2000), food waste (Xu et al. 2014; Zhen et al. 2020), lignocellulosic material (Nguyen et al. 2019; Xu et al. 2021), sewage sludge (Montalvo et al. 2016, 2018; Huiliñir et al. 2017), and different wastewater. For example, Xu et al. (2021) demonstrated enhanced hydrolysis of corn stover and a 17% increase in methane yield when a micro-aeration pretreatment of the substrate was applied. This was achieved by mixing the substrate in open air through 12 h, during which the DO concentration was maintained between 0.1 and 1.0 mg L^−1^ (Xu et al. 2021). Similarly, Nguyen et al. (2019) reported improved digestibility of lignocellulosic biomass and greater carbon recovery in the form of biogas production alongside enhanced system stability during intermittent micro-aeration in AD of Napier grass. The implementation of micro-aeration increased VS reduction from 21 to 47% and rapidly decreased VFA concentrations, reversing the poor performance observed under strict anaerobic conditions (Nguyen et al. 2019). Nonetheless, improved hydrolysis does not always lead to higher methane yields. For example, Botheju et al. (2010) reported a decrease in methane production from a synthetic complex wastewater after applying intermittent micro-aeration. The authors suggested that this decline could be attributed to over-aeration, which may have led to the oxidation of organic matter and diversion of electrons towards oxygen reduction instead of methanogenesis. Similarly, Johansen and Bakke (2006) verified that semi-continuous micro-aeration used as a pretreatment of primary sludge increased the hydrolysis but lead to a reduction in the methane yield. This reduction was primarily due to the oxidation of hydrolyzed products into carbon dioxide and their incorporation into new biomass, rather than being converted into methane. Although not reported in micro-aeration AD studies, another possibility for a lower methane production is the accumulation of intermediary compounds, derived from the hydrolysis of macromolecules, that can be inhibitory for syntrophic and methanogenic microorganisms, namely VFA, LCFA, and ammonia (Cirne et al. 2007; Cavaleiro et al. 2013). This could trigger the choice for a two-phase system, in which hydrolysis could be improved in the first micro-aerated step, while preventing inhibition of the microbial communities by the hydrolysis products in the second anaerobic step.

In general, applying micro-aeration effectively increases the hydrolysis and acidogenesis of the different substrates tested (Table S1), which has led to the choice of this strategy when targeting fatty acid production (Jagadabhi et al. 2010; Sawatdeenarunat et al. 2017; Cao et al. 2022; Duarte et al. 2024). For example, Duarte et al. (2024) studied the fatty acid production in AD of wastewater with high salinity content (20 g L^−1^). Under micro-aeration (0.3 L L^−1^ day^−1^), higher concentrations of hexanoate (a fatty acid with high market value) were observed, and lower ORP values were attained in comparison with anaerobic conditions, indicating the presence of aerobic or facultative anaerobic microorganisms, which have consumed the oxygen. Also, Liu et al. (2024) studied the effect of nanobubbles on medium-chain fatty acid production in AD of cow manure. The results demonstrated that the addition of air nanobubble considerably raised the hexanoate concentration (by 55%, up to 15.1 g L^−1^), relatively to the control group without air nanobubbles. The relative abundance of bacteria from the *Bacillus* and *Caproiciproducens* genera, which are involved in chain elongation, increased in the experiments with air nanobubbles. Additional works, focused on the effect of micro-aeration on the hydrolytic stage of fermentation and carboxylate production, are reviewed in Magdalena et al. (2022).

Besides all these effects of micro-aeration, increased NH_4_-N, total nitrogen (TN), and COD removal have also been reported, as well as more stable pH and reduced sludge yield (Table S1).

Additionally, micro-aeration has been applied in bioreactors aimed at pollutant removal, namely H_2_S (Table S2), hydrocarbons, polyphenols, pharmaceuticals, azo dyes, and siloxanes (Table 2). H_2_S is toxic to methanogens in concentrations ranging from 50.0 to 220.0 mg L^−1^ (at pH 7–8) (Vu et al. 2022) and can be effectively reduced through biological oxidation by introducing controlled amounts of air or pure oxygen into the reactor, which promotes the activity of naturally occurring sulphide-oxidizing bacteria such as *Thiobacillus* sp., leading to the conversion of H_2_S into elemental sulphur and some thiosulphate (Díaz et al. 2010, 2011; Chen et al. 2017; Jeníček et al. 2017; Vu et al. 2022). Micro-aeration is widely applied for this purpose (Table S2), including in full-scale municipal wastewater treatment plants, e.g. by injecting air into the sludge recirculation line (Jeníček et al. 2017) or into the biogas recirculation (European Biogas Association 2022). In the study by Jeníček et al. (2017), air doses of 1–3% of biogas production resulted in a maximum decrease in methane content of no more than 2%, ensuring undisturbed methanogenesis and tolerable biogas dilution. Compared to other desulfurization methods, micro-aeration achieves high H_2_S removal efficiencies (up to 99%) at lower operational costs (Fdz.-Polanco et al. 2009; Ramos et al. 2014). Although many applications focus on in situ H_2_S removal (Table S2) rather than methane yield, optimizing oxygen administration can ensure both effective desulfurization and methane production.

Some authors have extended the Anaerobic Digestion Model No. 1 (ADM1) to include sulphate reduction and sulphide oxidation processes, aiming to optimize the micro-aeration process during the anaerobic treatment of sulphate-rich wastewater. This extended model, called ADM1-S/O, enables the optimization of both the oxygen intake and the micro-aeration initiation to reduce the H_2_S production in wastewater treatment (Pokorna-Krayzelova et al. 2017). Another modification of ADM1 model, ADM1-Ox, was introduced by Botheju et al. (2009), to describe the effect of free oxygen on anaerobic digestion. Model simulations, supported by experimental data, suggested a linear reduction in the cumulative methane production potential from glucose as oxygen concentrations increased. However, the O_2_ concentrations tested (22, 44, and 88 mg L^−1^) may exceed the typical range associated with micro-aeration. Nevertheless, the ADM1-Ox remains a valuable modelling framework for investigating the influence of low oxygen levels on anaerobic digestion and could be further adapted to reflect more realistic micro-aerobic conditions. In most of the applications regarding pollutant removal (other than H_2_S), air (and not pure oxygen) has been generally used, frequently applied intermittently (Table 2). Wu and colleagues investigated the effect of micro-aeration as a pretreatment of petrochemical wastewater before anaerobic treatment. Their results demonstrated that limited aeration, with a DO concentration of 0.2–0.3 mg L^−1^ and an average ORP of − 210 mV, almost eliminated H_2_S generation in the off-gas when DO was higher than 0.2 mg L^−1^. Additionally, the specific oxygen uptake rate of the petrochemical wastewater increased, indicating a substantial reduction in effluent toxicity (Wu et al. 2015). In a full-scale system, the same authors showed that implementing limited aeration in a hydrolysis acidification tank (as a pretreatment before the anoxic tank) effectively degraded 49 micropollutants in petrochemical wastewater, including BTEX (benzene, toluene, ethylbenzene, and xylene) compounds and phenols (Table 2). The micro-aerobic hydrolysis and acidification process increased the COD removal from 11% (in traditional anaerobic hydrolysis) to 25% (Fig. 2), while the BOD_5_/COD ratio improved from 0.23 to 0.43, indicating enhanced biodegradability. Additionally, the process reduced the formation of H_2_S and improved micro-aerobic degradation, with the remaining COD after 72 h dropping to less than 80.0 mg L^−1^, compared to over 100.0 mg L^−1^ in untreated effluent. This study demonstrated that the integrated process achieved stable performance and significantly improved effluent quality (Wu et al. 2018).

Similarly, Carvalho et al. (2020) reported that a micro-aerobic environment was more effective in reducing aromatic amines formed during the anaerobic digestion of textile wastewater containing the dye Direct Black 22 (Table 2). In another application, Ortiz-Ardila et al. (2024) demonstrated that micro-aeration enhanced the biodegradation of volatile siloxanes (octamethylcyclotetrasiloxane and decamethylcyclopentasiloxane) in wastewater sludge (Table 2). This process promoted their conversion into methane and simpler monomeric compounds, such as phenyl-siloxanes, linear siloxanes, and tripropyl-silanes, which were identified as metabolic intermediates. These changes reshaped microbial communities and improved overall process efficiency (Ortiz-Ardila et al. 2024).

**Fig. 3:**
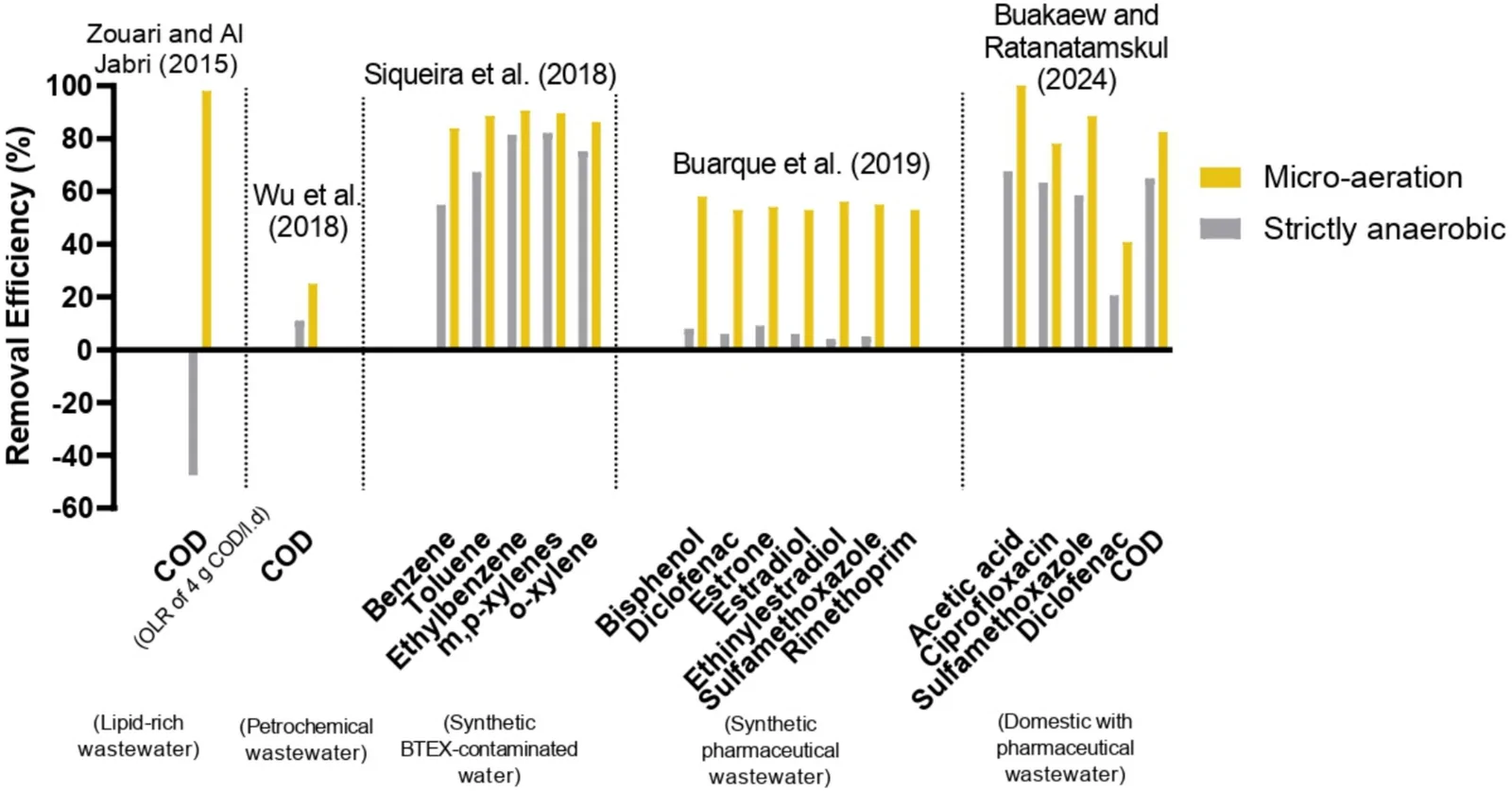
Comparison of removal efficiency for various pollutants under micro-aeration and anaerobic treatment

Buarque et al. (2019) showed that, by applying micro-aeration (1.0 mL min^−1^, equivalent to a ratio between the air and wastewater flow rate of 0.1), the process significantly enhanced the removal of various emerging micropollutants, including natural and synthetic estrogens (estrone (E1), estradiol (E2), and ethinylestradiol (EE2)), pharmaceuticals (diclofenac (DCF), sulfamethoxazole (SMX), and trimethoprim (TMP)), and the xenoestrogen bisphenol A (BPA) (Fig. 3). Under anaerobic conditions, the removal efficiencies were very low (< 10%), with specific values of 9% for E1, 6% for E2, 4% for EE2, 6% for DCF, 5% for SMX, − 4% for TMP, and 8% for BPA. However, with the introduction of micro-aeration, the removal efficiencies increased to 54% for E1, 53% for E2, 56% for EE2, 53% for DCF, 55% for SMX, 53% for TMP, and 58% for BPA. Buakaew and Ratanatamskul (2024) investigated the performance of a novel anaerobic baffled biofilm-membrane bioreactor (AnBB-MBR) for enhancing pharmaceutical removal from domestic wastewater. The higher ciprofloxacin and diclofenac removals (78% and 41%, respectively) were attained when the bioreactor was operated with micro-aeration (DO in the bioreactor in the range of 0.3–0.7 mg L^−1^ and oxygen supply rate at 0.93 L L^−1^ (volume of O_2_ per volume of feed)). Sulfamethoxazole was also successfully removed in the bioreactor operated with micro-aeration (89%) (Fig. 3) and increased further when micro-aeration was combined with sludge recirculation (91%).

In another case, micro-aeration was used to pretreat olive mill wastewater (OMWW) to facilitate the breakdown of polyphenols. An oxygenation rate of 0.13 L L^−1^ day^−1^ over 5 days led to reductions in total COD and increased methane yield, producing a more stable AD process. The authors concluded that anaerobic digestion of OMWW could be economically feasible if pretreated aerobically (González-González and Cuadros 2015).

### Micro-aeration for enhancing lipid conversion in AD

Besides the pollutants already discussed, micro-aeration has also been applied to improve lipid degradation in AD and their conversion to methane. This topic has not been reviewed before in the literature, and thus, it will be addressed here in a separate section.

Lipids are among the most valuable substrates for producing biomethane since their theoretical potential represents 1.425 L of biogas per g of substrate, with methane accounting to 70%, while other carbon sources like proteins and carbohydrates only achieve 0.921 L g^−1^ (69% of CH_4_) and 0.830 L g^−1^ (50% of CH_4_), respectively (Alves et al. 2009). In the AD process, the hydrolysis of lipids results in the generation of long-chain fatty acids (LCFA). Usually, lipids and LCFA are often separated from wastewater previously to AD processes, since their presence is related to operational issues mostly triggered by LCFA accumulation on the sludge, which causes biomass flotation and washout. Moreover, LCFA have been reported as toxic or inhibitory to the anaerobic microbial communities and particularly to methanogens (Hanaki et al. 1981; Angelidaki and Ahring 1992; Rinzema et al. 1994). LCFA tend to accumulate onto the sludge, creating a physical barrier and delaying the transfer of substrates and products, which further results in a delay on the methane production (Pereira et al. 2005).

In the last decades, several approaches have been proposed to overcome the LCFA/lipid toxicity, as reviewed by Holohan et al. (2022), such as bioaugmentation with LCFA-degrading bacteria (Cavaleiro et al. 2010), sludge acclimatization to LCFA (Silva et al. 2014; Ziels et al. 2016), feeding strategies (Cavaleiro et al. 2009; Ziels et al. 2017), addition of activated carbon (Tan et al. 2021) or adsorbents like bentonite (Palatsi et al. 2012), and precipitation with calcium salts (Hanaki et al. 1981). Despite previous research efforts made on this topic (Alves et al. 2009; Holohan et al. 2022), the amount of lipids effectively converted into methane is still limited. Recently, micro-aeration was shown to be a promising approach for optimizing AD of lipids/LCFA.

**Fig. 4:**
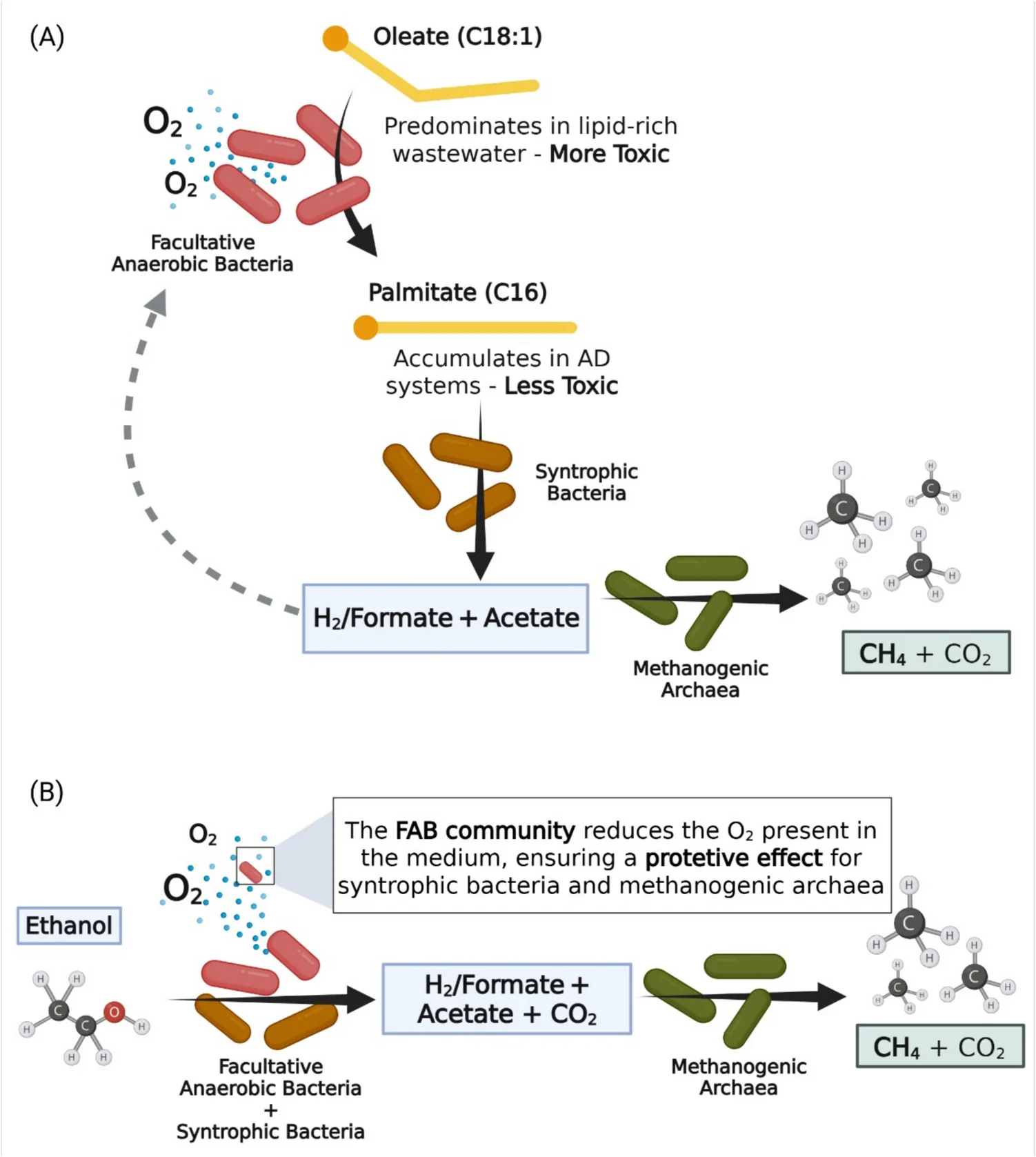
Proposed mechanistic scheme for the degradation of lipids and other compounds in syntrophic anaerobic systems under micro-aeration conditions. **A** Oleate-to-palmitate conversion by facultative anaerobic bacteria (FAB), followed by the syntrophic conversion of palmitate into H_2_/formate and acetate coupled to methane production by methanogenic archaea. **B** Ethanol oxidation (as model substrate for syntrophic communities) by FAB, alongside with anaerobic degradation by syntrophic bacteria coupled to methane production by methanogenic archaea. Created in BioRender. Costa, L. (2025) https://BioRender.com/2k3jrut

Oleate (C18:1 LCFA) is the most prevalent LCFA found in wastewaters (Baserba et al. 2012) and therefore is often used as model compound. However, when oleate is fed in continuous bioreactors, palmitate (C16:0) is the main LCFA that accumulates (Pereira et al. 2002). Duarte et al. (2018) showed that palmitate accumulates more in the presence of low oxygen concentrations. Moreover, contrary to what was previously thought, palmitate accumulation was shown to be advantageous for methane production (Duarte et al. 2018), avoiding complete inhibition of methanogens, possibly because palmitate is less toxic than oleate. The abundance of facultative anaerobic bacteria (FAB), namely *Pseudomonas* spp., was strongly correlated with the ratio between palmitate and total LCFA (Cavaleiro et al. 2016; Duarte et al. 2018) and to more efficient methane production from oleate (Duarte et al. 2018; 2020). Thus, these works suggest that micro-aeration can trigger a first treatment step that converts oleate to palmitate (Fig. 4A), which facilitates further LCFA degradation to methane and supports anaerobic treatment of lipid-rich wastes/wastewaters. Nevertheless, the mechanisms underlying the oleate-to-palmitate conversion, as well as the role of FAB on this conversion, are still not fully understood.

In the follow-up of this work, the *Pseudomonas* spp. previously isolated by Duarte et al. (2018) were studied together with a syntrophic co-culture (*Syntrophomonas zehnderi* and *Methanobacterium formicicum*), in a continuous bioreactor treating oleate-based wastewater, under micro-aerobic conditions (O_2_ provided in the feeding) (Duarte et al. 2020). The results showed that the *Pseudomonas* spp. could also act as alternative hydrogen/formate scavengers, favouring the activity of syntrophic LCFA-degrading bacteria while shielding the strict anaerobic community from oxygen toxicity (Fig. 4A). More recently, the work of Magalhães et al. (2025) studied the performance of synthetic syntrophic co-cultures involved in the degradation of short-, medium-, and long-chain fatty acids, under micro-aerobic conditions in batch bioreactors. The syntrophic activity was reduced by 79% when 0.5% O_2_ (v/v in the headspace) was applied, whereas syntrophic co-cultures amended with the *Pseudomonas* spp. isolated by Duarte et al. (2018) were able to convert the fatty acids to methane at O_2_ concentrations up to 2% (v/v in the headspace).

These results were also confirmed by the work of Morais et al. (2024) that studied the effect of oxygen (0 to 5% v/v in the headspace) on the syntrophic activity of a complex methanogenic community (anaerobic sludge). By using specific substrates of methanogenesis (H_2_/CO_2_ and acetate), these authors showed that oxygen inhibited both hydrogenotrophic and aceticlastic methanogens, resulting in 30–40% inhibition of the methane production rate (MPR) already at 0.5% O_2_, relatively to the controls (0% O_2_). However, in cultures amended with ethanol (used as a model substrate for the syntrophic community), the inhibition was significantly lower, with only 15% reduction in the MPR at 0.5% O_2_. Moreover, in the assays with ethanol, MPR inhibition similar to the value observed for methanogens (i.e. 36 ± 7%) was only verified at 2.5% O_2_. This demonstrates the higher resilience of ethanol-amended cultures to oxygen toxicity, likely due to the activity of FAB and aerobic bacteria, which consumed the ethanol and oxygen thus protecting the methanogenic community (Fig. 4B) (Morais et al. 2024).

All these works highlight that FAB are crucial for the syntrophic metabolism under micro-aerobic conditions, by protecting microbial consortia from potential oxygen damage. (Stams and Plugge 2009; McInerney et al. 2009) Additionally, Magalhães et al. (2025) also highlighted that this protective effect was maintained during repeated micro-aerobic exposure. Given that the majority of large-scale AD plants function in non-strict anaerobic environments, the presence of these bacteria is crucial for preserving robust, stable, and functional syntrophic communities. Nevertheless, it is worth noting that micro-aeration does not always improve the methane production rate comparatively to anaerobic conditions, which points that the activity of FAB may be more relevant for the degradation of challenging substrates or when the microbial communities are not adapted to the toxic substrates (Magalhães et al. 2025).

A recent work from Li et al. (2025) used the principles of the biochemical methane potential (BMP) test, with acetate and glucose as substrates, to study the best micro-aeration intensity (expressed as the volume of air per mass unit of the substrate in COD) for methanogenesis. Micro-aeration intensities ranged from 0 to 120 mL g^−1^ for aceticlastic methanogenesis (using acetate as the substrate) and from 0 to 60 mL g^−1^ for the overall glucose conversion to methane. To make the results comparable with other studies, these values correspond to 0 to 8% O_2_ (v/v in the headspace) for both situations. The results pointed to 30 mL g^−1^ (2% O_2_) and 7.5 mL g^−1^ (1% O_2_) as the best micro-aeration intensities for acetate and the overall glucose conversion to methane, respectively. Using these micro-aeration intensity values, experiments were then performed in the presence of 0.5 mmol L^−1^ oleate, which increased the total amount of methane by 22% in the assays with acetate but inhibited the methane production by 17% in assays with glucose. This study found that FAB, including those from the genera *Pseudomonas*, *Actinomyces*, and *Alcaligenes*, thrived more quickly at intermediate micro-aeration intensities. While the abundance of the genus *Methanosaeta* fell by 65% in the assays with glucose, the abundance of overall methanogenic archaea increased by 27% in the assays with acetate. Therefore, to maximize the effectiveness of methanogenesis, the proper micro-aeration intensity should be systematically considered.

Despite limited studies in this area, preliminary lab-scale investigations suggest that micro-aeration may help address the challenges in AD when applied as a pretreatment for lipid-rich wastes. For example, Zouari and Al Jabiri (2015) found that mesophilic micro-aerobic pretreatment (0.05 volume of air per volume of feeding solution per minute, vvm) of lipid-rich slaughterhouse wastewater reduced total suspended solids (TSS) by 20%, while under strict anaerobic conditions, TSS accumulated in the reactor, accompanied by a decrease in methane production. This work also showed that micro-aeration improved methane yields by 19 and 20% in comparison with strict anaerobic conditions, regarding soluble and total COD, respectively (Zouari and Al Jabiri 2015). However, higher oxygenation rates (0.15 vvm) inhibited methanogenesis, demonstrating the need for precise control.

In conclusion, by applying micro-aeration in AD of lipids, FAB growth and activity are stimulated, which promotes LCFA conversion to methane and improves the overall anaerobic treatment of the compounds in wastes/wastewaters. Therefore, micro-aeration has the potential to evolve into a crucial tool for enhancing the efficiency of the anaerobic digestion of lipids/LCFA in methanogenic reactors.

## Conclusions and future perspectives

Despite its rowing relevance as a renewable energy source, biomethane production from wastewater still faces significant challenges. Overcoming these limitations is essential to enhance process efficiency and scalability. While notable progress has been made using micro-aeration, several challenges remain unresolved. In particular, the application of micro-aeration in AD for the removal of toxic and recalcitrant organic pollutants, as well as their conversion to methane, has lagged behind its use in other areas, highlighting the need for further research. Main challenges hindering the practical application of micro-aeration are highlighted as follows:

1. Defining optimal micro-aeration intensity: excessive oxygen can inhibit the activity of strict anaerobic microorganisms and divert carbon flux from methane production to aerobic metabolism. Conversely, insufficient oxygen can yield negligible or negative effects on bioreactor performance. This complexity arises from multiple factors, including the microbial community’s composition, its adaptation to toxic compounds, and tolerance to oxygen. Developing robust mathematical models, integrated with machine learning and/or other artificial intelligence (AI) tools, could pave the way for a data-driven decision system. In addition, multi-stage reactors can be designed, with dedicated zones for micro-aeration, to handle varying pollutant loads and also to better manage the susceptibility to oxygen of the various microbial groups in AD. Digital twins can also be implemented to simulate and optimize micro-aeration.
2. Understanding mechanisms and microbial interactions: a deeper understanding of microbial community dynamics, metabolic pathways, and cellular communication systems, underlying the response to micro-aerobic conditions and prompting microbial interactions, is essential. High-throughput sequencing techniques can contribute to deepen this knowledge, which will enable the fine-tuning of micro-aeration processes to achieve desired outcomes, enhancing reaction rates and methane yields. Furthermore, this knowledge can also prompt the development of synthetic microbial consortia, optimized to target specific pollutants under micro-aerobic conditions. Additionally, the kinetics of the biodegradation of organic pollutants, promoted by micro-aeration, should also be investigated.
3. Addressing oxygen mass transfer limitations and process control: despite advancements, oxygen mass transfer limitations and oxygen losses in biogas remain far from optimal. Further improvements in these areas are necessary to enhance the efficiency of micro-aeration systems. Moreover, automatic process control remains a bottleneck for the large-scale implementation of micro-aeration-based AD systems. Designing a reliable, automatically controlled, and long-term stable micro-aeration dosing system is pivotal for the widespread application of this technology. Additionally, current methods for measuring oxygen provide only momentary concentration data, lacking the ability to track oxygen flow, or the reactions it undergoes. Developing more comprehensive measurement tools will be essential for improving process monitoring and control.

The application of micro-aeration to enhance the degradation of recalcitrant pollutants in AD is still in its early stages and represents an opportunity to be explored, particularly regarding emerging contaminants that remain largely unexplored, such as, for example, microplastics and persistent organic pollutants (POPs). Moreover, synergies with advanced pre- and posttreatment strategies can be explored to further improve the breakdown of these complex organic compounds. While aeration introduces additional energy costs, these can be mitigated by using renewable energy sources (namely biogas/biomethane for electricity generation to drive aeration turbines). Importantly, the cost-efficiency of the process should be evaluated considering the environmental value of pollutant removal, which is often overlooked due to the difficulty of assigning a direct economic figure. This framing helps position micro-aeration as both an environmental and potentially economically viable strategy to improve AD.

## Supplementary information

## Supplementary information

Below is the link to the electronic supplementary material.

ESM 1 (PDF 297 KB)
