# Supplementary material for: Micro-aeration for improving anaerobic treatment and biogas production from organic pollutants
Source: Appl Microbiol Biotechnol. 2025 May 30;109:131. doi: 10.1007/s00253-025-13519-w (PMC12125087; doi:10.1007/s00253-025-13519-w)
Supplement: Supplementary file 1 [file 253_2025_13519_MOESM1_ESM.pdf]

Micro-Aeration for Improving Anaerobic Treatment and Biogas Production from Organic Pollutants

Luís Costa[1], M. Salomé Duarte[1,2,], Carla P. Magalhães[1], M. Alcina Pereira[1,2], Ana J. Cavaleiro[1,2#]

[1] CEB - Centre of Biological Engineering, University of Minho, Campus de Gualtar, Braga, Portugal

[2] LABBELS – Associate Laboratory, Braga/Guimarães, Portugal

# Corresponding author: A.J. Cavaleiro, acavaleiro@ceb.uminho.pt

Luís Costa, id11211@alunos.uminho.pt, ORCID: 0000-0001-6347-640X

Maria Salomé Duarte, salomeduarte@ceb.uminho.pt, ORCID: 0000-0003-4645-908X

Carla Pereira Magalhães, c.pereira.mg@gmail.com, ORCID: 0000-0002-4763-4989

Maria Alcina Pereira, alcina@deb.uminho.pt, ORCID: 0000-0002-7110-1779

Ana Júlia Cavaleiro, acavaleiro@ceb.uminho.pt, ORCID: 0000-0002-7999-4620

Table S1 Summary of studies that applied micro-aeration on anaerobic digestion for improving the hydrolysis, including key parameters such as substrate, reactor configuration, air/oxygen dosage, and observed effects

Substrate	Reactor type	Air/O_2	Pre-treatment (Pt) Direct (D)	Air/O_2 Dose	Effect	Reference
Primary sludge	Batch bottles	Air	Pt - Semi-continuous (time-based)	0.21 L L^{-1} d^{-1}	Increased hydrolysis (50-60%); Reduced CH_4 yield	(Johansen and Bakke, 2006)
Primary sludge	Batch Flasks	Air	D - Continuous	0.5 L L^{-1} d^{-1}	Increased hydrolysis	(Diak et al. 2013)
40% primary sludge and 60% WAS	CSTR	Air	D - Pulses	12.5 and 25 mL L^{-1} d^{-1}	Increased biogas production (16±7%)	(Zhou et al. 2021)
WAS	CSTR	Air	D - Continuous	24-121 L L^{-1} d^{-1}	Increased solubilization	(Hasegawa et al. 2000)
WAS	CSTR	Air	D - Continuous	0.03 L L^{-1} d^{-1}	Decreased VFA; Decreased foaming; Better dewaterability	(Jenicek et al. 2014)
Mixed WAS and fly ashes	Batch bottles	Air	Pt - Continuous (48h)	0.35 vvm for 48 h	Increased CH_4 production (70%)	(Montalvo et al. 2018)
Wastewater primary sludge and WAS	CSTR	Air	D - Continuous (4h)	0-6 Air volume g^{-1} TS min^{-1}	Increased VFA; Increased biogas production (16%)	(Ruan et al. 2019)
Mixed sewage sludge	Reactor	Air	Pt - Continuous (48h)	0.3 vvm	Increase hydrolysis; Increased biogas	(Montalvo et al. 2016)
Sewage sludge	Batch bottles	Air	Pt - Semi-continuous	0.1, 0.2, 0.3 and 0.5 vvm (20h)	VSS reduction (31%); Increased microbial activities (597 %); Increased CH_4 production (221 %)	(Barati rashvanlou et al. 2020)
Activated sludge	AnMBR	Air	D - Continuous (ORP based)		Increased CH_4 production; Improved sludge filterability	(Wei et al. 2024)

Table S1 (cont.)

Substrate	Reactor type	Air/O_2	Pre-treatment (Pt) Direct (D)	Air/O_2 Dose	Effect	Reference
Synthetic wastewater with sucrose	Bench scale reactors	Air	D - Semi-continuous (time-based)	0.07 and 0.7 L L^{-1} d^{-1}	Increased COD removal; More stable pH	(Zitomer and Shrout 1998)
Synthetic wastewater (starch, peptone, yeast extract, phosphate salts)	Batch reactor	Air	D - Intermittent (daily pulses)	0, 2.52, 5.04 and 10.07 (as % of feed COD)	Decrease of CH_4 /biogas; Decrease of VFA accumulation; More stable operation	(Botheju et al. 2010)
Wastewater treatment plant	Pilot scale bioreactors	Air	Pt - Continuous	DO level of 3.0-4.0 mg L^{-1}	Decrease sludge yield	(Niu et al. 2016)
Industrial wastewater and domestic sewage	Full scale anaerobic–anoxic–aerobic with a microaerobic tank	Air	D - Continuous	0.5-1.0 mg L^{-1}	Low sludge yield (0.074 g SS g^{-1} COD); High NH_4-N, TN and COD removal	(Jiang et al. 2018)
Lipid rich slaughterhouse wastewater	Bottles	Air	Pt - Continuous (through feeding)	0.05 vvm and 0.15 vvm	Reduced TSS; Increased digestibility; Increased CH_4 production yields	(Zouari and Al Jabiri, 2015)
Brown water and food waste	Batch reactors	Air	Pt - Continuous	0.04 L L^{-1} d^{-1}	Increased hydrolysis and acidogenesis; Increased CH_4 (21%)	(Lim and Wang, 2013)
Brown water and food waste	CSTR	O_2	D - Single dosing	0.005 and 0.007 L L^{-1} d^{-1}	Increased hydrolysis; Increased acidogenesis	(Lim et al. 2014)
Food waste	Semi-continuous digester	Air	D- Semi-continuous	5 mL air per feeding (corresponding to an increase of 30–50 mV in ORP)	Enhanced CH_4 yield; Improved system stability; Increased microbial activity; Reduced dissolved organic matter	(Ding et al. 2024)

Table S1 (cont.)

Substrate	Reactor type	Air/O_2	Pre-treatment (Pt) Direct (D)	Air/O_2 Dose	Effect	Reference
Kitchen waste	SBR	O_2	Pt - Single dosing	1.11 mL g^{-1} VS d^{-1})	Increased biogas production	(Zhen et al. 2020)
Grass silage	Leach bed reactor	Air	D - Semi-continuous (time-based, 24h)	0.5 and 5 L L^{-1} d^{-1}	Increased VFA production (4x); Extensive aeration: VFA reduction	(Jagadabhi et al. 2010)
Napier grass	Batch bottles	O_2	D - Single dosing	15 mL gVS^{-1} = 0.09 and 1.9 L L^{-1} d^{-1}	Increased VFA production	(Sawatdeenarunat et al. 2017)
Napier grass	CSTR	O_2	C - Intermittent (every 24h) (ORP-based)	0.2 L L^{-1} d^{-1}	No VFA accumulation; Increased biogas production	(Nguyen, et al., 2019)
Corn straw	Batch bottles	O_2	Pt - Single dosing	5 mL g^{-1} VS^{-1} substrate	Increased hydrolysis; Increased CH_4 yield (16 %); Reduced lag phase	(Fu et al. 2015)
Corn straw	Batch bottles	O_2	Pt - Single dose	5 mL O2 g^{-1} VS	Increased CH_4 yield (271 mL g^{-1} VS); Increased glucose and VFA production	(Xu et al., 2018)
Corn straw	Batch bottles	O_2	Pt - Nanobubbles		Increased lignocellulosic hydrolysis (20-38%); Increased CH_4 yield (10-20%)	(Wang, et al., 2020) b
Corn derived wastewater (nejayote) + GAC	2 stage system APBR UASB	Air	D - Intermittent (time-based)	0.7 L min^{-1}	Increased VFA production (62%); Increased CH_4 yield (55%)	(Valero et al. 2020)
Dry corn stover	NM	Air	Pt -Continuous - Feeding open to air	0.1-1 mg L^{-1}	Increased hydrolysis; Increased AD rate	(Xu et al. 2021)
Wheat straw	Batch reactor	O_2	D - Intermittent (time-based)	5 mL O_2 g^{-1} VS	Increased hydrolysis; Increased Biogas	(Tsapekos et al. 2017)

Table S1 (cont.)

Substrate	Reactor type	Air/O_2	Pre-treatment (Pt) Direct (D)	Air/O_2 Dose	Effect	Reference
Wheat straw	Batch reactor	O_2	D - Intermittent (time-based)	5 mL O_2 g^{-1} VS	Increased hydrolysis; Increased Biogas	(Tsapekos et al. 2017)
Municipal solid waste	Pilot scale Leach bed reactor	Air	D - Semi-continuous (time-based)	0.39 L L^{-1} d^{-1}	Increased CH_4 production Increased hydrolysis and acidification	(Nguyen et al. 2007)
Municipal solid waste	Landfill simulation reactors		D - Intermittent	0.025 L $h^{-1}g^{-1}$, 0.05 L h^{-1} kg^{-1}, and 0.1 L h^{-1} kg^{-1}	Increased degradation of VFA; NH_4^+-N and organic matter reduction	(Liu et al. 2024)
Synthetic food waste	Leach bed rector	Air	D - Semi-continuous (time-based)	2.1, 4.4 and 6.5 L L^{-1} d^{-1}	Increased carbohydrate hydrolysis (21-27%); Increased protein hydrolysis (38-64%); Increased CH_4 yield	(Xu et al., 2014)
Cellulose	Batch bottles	Air	D - Nanobubbles		Increased VFA production; Increased CH_4 yield (10-18%)	(Wang, et al., 2020)
Cellulose	Batch bottles	Air<br>O_2	Pt - Nanobubbles	Air- NBW (193 NmL g^{-1} VSreduced)<br>O_2-NBW (233 NmL g^{-1} VSreduced)	Reduced cellulose crystallinity; Increased cellulase production Increased CH_4 yield (8-30%);	(Wang et al. 2020c)
Paper waste	Batch bottles	O_2	Pt - Single dosing	0, 5, 15, and 30 mL g^{-1} VS	Increased hydrolysis and acetogenesis; Increased CH4	(Song et al. 2021)
Cow manure	Batch bottles	Air	D - Pulses	37.5 mL L^{-1} min^{-1}	Increased hydrolysis	(Li et al. 2023)
Fresh 'as excreted' buffalo manure	Batch bottles	Air	Pt - Single dosing	7.3 mL g^{-1} VS (12h)	Increased lignin degradation	(Zeb et al. 2022)
1) Swine manure 2) corn silage + swine manure	Batch	Air	D - Intermittent	0.2 L L^{-1} d^{-1}	Higher VFA; lower lag phase	(Cao et al. 2022)

COD, Chemical oxygen demand. CSTR, Continuous stirred tank reactor. GAC, Granulated activated carbon. NM, Not mentioned. WAS, waste activated sludge. AnMBR, Anaerobic membrane bioreactor. APBR, Anaerobic packed bed reactors. NBW, Nanobubble water. SBR, Sequencing batch reactor. UASB, up-flow anaerobic sludge blanket. VFA, volatile fatty acids. VS, volatile solids. VSS, volatile suspended solids. vvm, gas volume flow per unit of liquid volume per minute.

Table S2 Summary of studies that applied micro-aeration on anaerobic digestion for H_2S removal, including key parameters such as substrate, reactor configuration, air/oxygen dosage, and observed effects

Substrate	Reactor type	Air/O_2	Pre-treatment (Pt) Direct (D)	O_2 Dose	Effect	Reference
Primary sludge	CSTR	O_2	D - Semi-continuous (ORP-based)	0.14 mL s^{-1}	Removal of H2S (99 %) No effects in VS and COD removal	(Nghiem et al. 2014)
WAS	Pilot scale bioreactor	Air/ O_2	D - Continuous (sludge recirculation)	0.25 L L^{-1} d^{-1}	Removal of H2S (98%) COD removal, VS reduction ad CH4 yield not affected	(Díaz et al. 2010)
WAS	Pilot scale CSTR	O_2	D - Continuous	0.013-0.024 L L^{-1} d^{-1}	Removal of H2S (99% Without negative effects on CH4	(Fdz.-Polanco et al. 2009)
WAS	Pilot scale bioreactor	O_2	D - Continuous (Biogas, sludge recirculation or headspace)	0.01 L L^{-1} d^{-1}	Removal of H2S (98%)	(Díaz et al. 2011)
WAS	Pilot scale CSTR	O_2	D - Continuous	0.01 L L^{-1} d^{-1}	Overcome overloading; Removal of H2S (90%); Prevention of severe imbalance	(Ramos and Fdz-Polanco, 2013)
Sewage sludge	Pilot scale CSTR	O_2	D - Continuous (headspace, biogas recirculation, liquid)	0.002 %-0.20 % (v/v)	High removal of H2S No difference in the digestion Increase of community diversity	(Ramos et al. 2014)
Pulp mill wastewater	UASB	Air	D - Continuous (in the feeding immediately entering the reactor)	0.9-1.8 L L^{-1} d^{-1}	Removal of H2S (30%) Increased COD removal rate (50%)	(Zhou et al., 2007)
High solid wastewater	CSTR + SOU	Air	D - Continuous (ORP-based)	0.0782 L L^{-1} d^{-1}	Removal of H2S (98%)	(Duangmanee et al. 2007)
Synthetic wastewater with ethanol	Continuous-flow anaerobic reactors	Air	D - Continuous	0.2 mL min^{-1}	Reduced H2S (93%) Increased system stability	(Sousa et al. 2016)
Municipal wastewater	EGSB	Air	D - Sludge recirculation	DO of 0.2 mg L^{-1}	Removal H2S (70%)	(Chen et al. 2017)

Table S2 (cont.)

Substrate	Reactor type	Air/O_2	Pre-treatment (Pt) Direct (D)	O_2 Dose	Effect	Reference
Municipal wastewater	Full scale plants	Air	D - Sludge recirculation	0.056-1.2 m^3 h^{-1}	Removal H2S (74%-99%)	(Jeníček et al. 2017)
Sulfate-rich wastewater	Batch reactors	O_2	D - Continuous (Biogas recirculation, ORP based)	1-2 mL min^{-1}	Removal of H2S (>99) Increased COD removal (5 %); Increased CH4 rate production (56 %)	(Khanal and Huang, 2003)
Sulfate-rich wastewater	UAF	O_2	D - Continuous (Biogas recirculation, ORP based)	5-6 mL min^{-1}	Removal of H2S (>98.5 %) Increase CH4 rate production (46%) Airflow >25 mV CH4 negative impact	(Khanal et al. 2003)
Sulfate-rich wastewater	UAF	O_2	D - Semi-continuous (ORP-based)		Removal of H2S (<99%) Increased CH4 yield (46%)	(Khanal and Huang 2006)
Synthetic brewery wastewater	UASB	O_2	D - Continuous	0.08 L L^{-1} d^{-1}	Removal of H2S (73 %)	(Krayzelova et al. 2014)
Vinasse	Fluidized bed reactor	Air	D - Continuous	0.15-0.19 L L^{-1} d^{-1}	Removal of H2S (99%) Faster process	(van der Zee et al. 2007)
Cheese way	Pilot scale CSTR	Air	D - Continuous (through gas membrane)	0.8, 1.2, 2, 4, 40, 400 mL min^{-1}	Decrease of H2S (3000 ppm to <less than 100 ppm) COD not affected	(Pokorna-Krayzelova et al. 2018)
Cow manure	Full-scale digester	Air	D - Continuous (in the headspace)	1% (v/v) biogas production rate	Removal of H2S (68.2%)	(Kobayashi et al. 2012)

COD, Chemical oxygen demand. CSTR, Continuous stirred tank reactor. EGSB, expanded granular sludge bed. SOU, Sulfide oxidizing unit. UAF, Up-flow anaerobic filter. UASB, up-flow anaerobic sludge blanket. VS, volatile solids.

References:

Barati rashvanlou R, Rezaee A, Farzadkia M, Gholami M, Kermani M (2020) Effect of micro-aerobic process on improvement of anaerobic digestion sewage sludge treatment: flow cytometry and ATP assessment. RSC Adv 10:35718–35728. https://doi.org/10.1039/D0RA05540A

Botheju D, Samarakoon G, Chen C, Bakke R (2010) An Experimental Study on the Effects of Oxygen in Bio-Gasification- Part 2. Renew Energy Power Qual J 1:1598–1604. https://doi.org/10.24084/repqj08.732

Cao Q, Zhang W, Lian T, Wang S, Yin F, Zhou T, Zhang H, Zhu J, Dong H (2022) Roles of micro-aeration on enhancing volatile fatty acids and lactic acid production from agricultural wastes. Bioresour Technol 347:126656. https://doi.org/10.1016/j.biortech.2021.126656

Chen C, Zhang R-C, Xu X-J, Fang N, Wang A-J, Ren N-Q, Lee D-J (2017) Enhanced performance of denitrifying sulfide removal process at high carbon to nitrogen ratios under micro-aerobic condition. Bioresour Technol 232:417–422. https://doi.org/10.1016/j.biortech.2017.02.031

Diak J, Örmeci B, Kennedy KJ (2013) Effect of micro-aeration on anaerobic digestion of primary sludge under septic tank conditions. Bioprocess Biosyst Eng 36:417–424. https://doi.org/10.1007/s00449-012-0798-x

Díaz I, Lopes AC, Pérez SI, Fdz-Polanco M (2010) Performance evaluation of oxygen, air and nitrate for the microaerobic removal of hydrogen sulphide in biogas from sludge digestion. Bioresour Technol 101:7724–7730. https://doi.org/10.1016/j.biortech.2010.04.062

Díaz I, Pérez SI, Ferrero EM, Fdz-Polanco M (2011) Effect of oxygen dosing point and mixing on the microaerobic removal of hydrogen sulphide in sludge digesters. Bioresour Technol 102:3768–3775. https://doi.org/10.1016/j.biortech.2010.12.016

Ding K, Wu B, Wang Y, Xu L, Liu M, Xiang J, Chen Y, Gu L, Li J, Li L, He Q, Liu S (2024) Study on synergistic effect of carrier combined with micro-aeration on anaerobic digestion of food waste. Chem Eng J 498:155731. https://doi.org/10.1016/j.cej.2024.155731

Duangmanee T, Kumar S, Sung S (2007) MICRO-AERATION FOR SULFIDE REMOVAL IN ANAEROBIC TREATMENT OF HIGH-SOLID WASTEWATER: A PILOT-SCALE STUDY. Proc Water Environ Fed 2007:2748–2760. https://doi.org/10.2175/193864707787960152

Fdz.-Polanco M, Díaz I, Pérez SI, Lopes AC, Fdz.-Polanco F (2009) Hydrogen sulphide removal in the anaerobic digestion of sludge by micro-aerobic processes: pilot plant experience. Water Sci Technol 60:3045–3050. https://doi.org/10.2166/wst.2009.738

Fu S-F, Wang F, Yuan X-Z, Yang Z-M, Luo S-J, Wang C-S, Guo R-B (2015) The thermophilic (55°C) microaerobic pretreatment of corn straw for anaerobic digestion. Bioresour Technol 175:203–208. https://doi.org/10.1016/j.biortech.2014.10.072

Hasegawa S, Shiota N, Katsura K, Akashi A (2000) Solubilization of organic sludge by thermophilic aerobic bacteria as a pretreatment for anaerobic digestion. Water Sci Technol 41:163–169. https://doi.org/10.2166/wst.2000.0068

Jagadabhi PS, Kaparaju P, Rintala J (2010) Effect of micro-aeration and leachate replacement on COD solubilization and VFA production during mono-digestion of grass-silage in one-stage leach-bed reactors. Bioresour Technol 101:2818–2824. https://doi.org/10.1016/j.biortech.2009.10.083

Jenicek P, Celis CA, Krayzelova L, Anferova N, Pokorna D (2014) Improving products of anaerobic sludge digestion by microaeration. Water Sci Technol 69:803–809. https://doi.org/10.2166/wst.2013.779

Jeníček P, Horejš J, Pokorná-Krayzelová L, Bindzar J, Bartáček J (2017) Simple biogas desulfurization by microaeration – Full scale experience. Anaerobe 46:41–45. https://doi.org/10.1016/j.anaerobe.2017.01.002

Jiang L-M, Zhou Z, Cheng C, Li J, Huang C, Niu T (2018) Sludge reduction by a micro-aerobic hydrolysis process: A full-scale application and sludge reduction mechanisms. Bioresour Technol 268:684–691. https://doi.org/10.1016/j.biortech.2018.08.070

Johansen J-E, Bakke R (2006) Enhancing hydrolysis with microaeration. Water Sci Technol 53:43–50. https://doi.org/10.2166/wst.2006.234

Khanal SK, Huang J (2006) Online Oxygen Control for Sulfide Oxidation in Anaerobic Treatment of High-Sulfate Wastewater. Water Environ Res 78:397–408. https://doi.org/10.2175/106143006X98804

Khanal SK, Huang J-C (2003) ORP-based oxygenation for sulfide control in anaerobic treatment of high-sulfate wastewater. Water Res 37:2053–2062. https://doi.org/10.1016/S0043-1354(02)00618-8

Khanal SK, Shang C, Huang J-C (2003) Use of ORP (oxidation-reduction potential) to control oxygen dosing for online sulfide oxidation in anaerobic treatment of high sulfate wastewater. Water Sci Technol 47:183–189. https://doi.org/10.2166/wst.2003.0645

Kobayashi T, Li Y-Y, Kubota K, Harada H, Maeda T, Yu H-Q (2012) Characterization of sulfide-oxidizing microbial mats developed inside a full-scale anaerobic digester employing biological desulfurization. Appl Microbiol Biotechnol 93:847–857. https://doi.org/10.1007/s00253-011-3445-6

Li X, Deng L, Li F, Zheng D, Yang H (2023) Effect of air mixing on high-solids anaerobic digestion of cow manure: Performance and mechanism. Bioresour Technol 370:128545. https://doi.org/10.1016/j.biortech.2022.128545

Lim JW, Chiam JA, Wang J-Y (2014) Microbial community structure reveals how microaeration improves fermentation during anaerobic co-digestion of brown water and food waste. Bioresour Technol 171:132–138. https://doi.org/10.1016/j.biortech.2014.08.050

Lim JW, Wang J-Y (2013) Enhanced hydrolysis and methane yield by applying microaeration pretreatment to the anaerobic co-digestion of brown water and food waste. Waste Manag 33:813–819. https://doi.org/10.1016/j.wasman.2012.11.013

Liu K, Li W, Zhang D, Lv L, Zhang G (2024) Positive effects of appropriate micro-aeration on landfill stabilization: Mitigating ammonia and VFAs accumulation. Bioresour Technol 413:131483. https://doi.org/10.1016/j.biortech.2024.131483

Montalvo S, Huiliñir C, Ojeda F, Castillo A, Lillo L, Guerrero L (2016) Microaerobic pretreatment of sewage sludge: Effect of air flow rate, pretreatment time and temperature on the aerobic process and methane generation. Int Biodeterior Biodegradation 110:1–7. https://doi.org/10.1016/j.ibiod.2016.01.010

Montalvo S, Vielma S, Borja R, Huiliñir C, Guerrero L (2018) Increase in biogas production in anaerobic sludge digestion by combining aerobic hydrolysis and addition of metallic wastes. Renew Energy 123:541–548. https://doi.org/10.1016/j.renene.2018.02.004

Nghiem LD, Manassa P, Dawson M, Fitzgerald SK (2014) Oxidation reduction potential as a parameter to regulate micro-oxygen injection into anaerobic digester for reducing hydrogen sulphide concentration in biogas. Bioresour Technol 173:443–447. https://doi.org/10.1016/j.biortech.2014.09.052

Nguyen D, Wu Z, Shrestha S, Lee P-H, Raskin L, Khanal SK (2019) Intermittent micro-aeration: New strategy to control volatile fatty acid accumulation in high organic loading anaerobic digestion. Water Res 166:115080. https://doi.org/10.1016/j.watres.2019.115080

Nguyen PHL, Kuruparan P, Visvanathan C (2007) Anaerobic digestion of municipal solid waste as a treatment prior to landfill. Bioresour Technol 98:380–387. https://doi.org/10.1016/j.biortech.2005.12.018

Niu T, Zhou Z, Shen X, Qiao W, Jiang L-M, Pan W, Zhou J (2016) Effects of dissolved oxygen on performance and microbial community structure in a micro-aerobic hydrolysis sludge in situ reduction process. Water Res 90:369–377. https://doi.org/10.1016/j.watres.2015.12.050

Pokorna-Krayzelova L, Bartacek J, Theuri SN, Segura Gonzalez CA, Prochazka J, Volcke EIP, Jenicek P (2018) Microaeration through a biomembrane for biogas desulfurization: lab-scale and pilot-scale experiences. Environ Sci (Camb) 4:1190–1200. https://doi.org/10.1039/C8EW00232K

Ramos I, Fdz-Polanco M (2013) The potential of oxygen to improve the stability of anaerobic reactors during unbalanced conditions: Results from a pilot-scale digester treating sewage sludge. Bioresour Technol 140:80–85. https://doi.org/10.1016/j.biortech.2013.04.066

Ramos I, Pérez R, Reinoso M, Torio R, Fdz-Polanco M (2014) Microaerobic digestion of sewage sludge on an industrial-pilot scale: The efficiency of biogas desulphurisation under different configurations and the impact of O2 on the microbial communities. Bioresour Technol 164:338–346. https://doi.org/10.1016/j.biortech.2014.04.109

Ruan D, Zhou Z, Pang H, Yao J, Chen G, Qiu Z (2019) Enhancing methane production of anaerobic sludge digestion by microaeration: Enzyme activity stimulation, semi-continuous reactor validation and microbial community analysis. Bioresour Technol 289:121643. https://doi.org/10.1016/j.biortech.2019.121643

Sawatdeenarunat C, Sung S, Khanal SK (2017) Enhanced volatile fatty acids production during anaerobic digestion of lignocellulosic biomass via micro-oxygenation. Bioresour Technol 237:139–145. https://doi.org/10.1016/j.biortech.2017.02.029

Song C, Li W, Cai F, Liu G, Chen C (2021) Anaerobic and Microaerobic Pretreatment for Improving Methane Production From Paper Waste in Anaerobic Digestion. Front Microbiol 12. https://doi.org/10.3389/fmicb.2021.688290

Sousa MR, Oliveira CJS, Lopes AC, Rodríguez ER, Holanda GBM, Landim PGC, Firmino PIM, Dos Santos AB (2016) Technical, Economical, and Microbiological Aspects of the Microaerobic Process on H2S Removal for Low Sulfate Concentration Wastewaters. Appl Biochem Biotechnol 180:1386–1400. https://doi.org/10.1007/s12010-016-2174-9

Tsapekos P, Kougias PG, Vasileiou SA, Lyberatos G, Angelidaki I (2017) Effect of micro-aeration and inoculum type on the biodegradation of lignocellulosic substrate. Bioresour Technol 225:246–253. https://doi.org/10.1016/j.biortech.2016.11.081

Valero D, Rico C, Tapia-Tussell R, Alzate-Gaviria L (2020) Rapid Two Stage Anaerobic Digestion of Nejayote through Microaeration and Direct Interspecies Electron Transfer. Processes 8:1614. https://doi.org/10.3390/pr8121614

van der Zee FP, Villaverde S, García PA, Fdz.-Polanco F (2007) Sulfide removal by moderate oxygenation of anaerobic sludge environments. Bioresour Technol 98:518–524. https://doi.org/10.1016/j.biortech.2006.02.011

Wang X, Lei Z, Shimizu K, Zhang Z, Lee D-J (2020a) Improved methane production from corn straw using anaerobically digested sludge pre-augmented by nanobubble water. Bioresour Technol 311:123479. https://doi.org/10.1016/j.biortech.2020.123479

Wang X, Yuan T, Guo Z, Han H, Lei Z, Shimizu K, Zhang Z, Lee D-J (2020b) Enhanced hydrolysis and acidification of cellulose at high loading for methane production via anaerobic digestion supplemented with high mobility nanobubble water. Bioresour Technol 297:122499. https://doi.org/10.1016/j.biortech.2019.122499

Wang X, Yuan T, Lei Z, Kobayashi M, Adachi Y, Shimizu K, Lee D-J, Zhang Z (2020c) Supplementation of O2-containing gas nanobubble water to enhance methane production from anaerobic digestion of cellulose. Chem Eng J 398:125652. https://doi.org/10.1016/j.cej.2020.125652

Wei C-H, Wang Z-W, Dai J-H, Xiao K, Yu H-R, Qu F-S, Rong H-W, He J-G, Ngo HH (2024) Enhanced anaerobic digestion performance and sludge filterability by membrane microaeration for anaerobic membrane bioreactor application. Bioresour Technol 402:130787. https://doi.org/10.1016/j.biortech.2024.130787

Xu H, Li Y, Hua D, Zhao Y, Chen L, Zhou L, Chen G (2021) Effect of microaerobic microbial pretreatment on anaerobic digestion of a lignocellulosic substrate under controlled pH conditions. Bioresour Technol 328:124852. https://doi.org/10.1016/j.biortech.2021.124852

Xu S, Selvam A, Wong JWC (2014) Optimization of micro-aeration intensity in acidogenic reactor of a two-phase anaerobic digester treating food waste. Waste Manag 34:363–369. https://doi.org/10.1016/j.wasman.2013.10.038

Xu W, Fu S, Yang Z, Lu J, Guo R (2018) Improved methane production from corn straw by microaerobic pretreatment with a pure bacteria system. Bioresour Technol 259:18–23. https://doi.org/10.1016/j.biortech.2018.02.046

Zeb I, Yousaf S, Ali M, Yasmeen A, Khan AZ, Tariq JA, Zhao Q, Abbasi AM, Ahmad R, Khalil TM, Yaqoob A, Bilal M (2022) In-situ microaeration of anaerobic digester treating buffalo manure for enhanced biogas yield. Renew Energy 181:843–850. https://doi.org/10.1016/j.renene.2021.09.089

Zhen X, Zhang X, Li S, Li M, Kang J (2020) Effect of micro-oxygen pretreatment on gas production characteristics of anaerobic digestion of kitchen waste. J Mater Cycles Waste Manag 22:1852–1858. https://doi.org/10.1007/s10163-020-01072-9

Zhou W, Imai T, Ukita M, Li F, Yuasa A (2007) Effect of limited aeration on the anaerobic treatment of evaporator condensate from a sulfite pulp mill. Chemosphere 66:924–929. https://doi.org/10.1016/j.chemosphere.2006.06.004

Zhou Z, Ming Q, An Y, Ruan D, Chen G, Wei H, Wang M, Wu Z (2021) Performance and microbial community analysis of anaerobic sludge digestion enhanced by in-situ microaeration. J Water Process Eng 42:102171. https://doi.org/10.1016/j.jwpe.2021.102171

Zitomer DH, Shrout JD (1998) Feasibility and benefits of methanogenesis under oxygen-limited conditions. In: Proceedings of the 52nd Purdue Industrial Waste Conference1997 Conference. CRC Press, pp 279–288

Zouari N, Al Jabiri H (2015) Improvement by Micro-Aeration of Anaerobic Digestion of Slaughterhouse Wastewater At 38 C. Int J Innov Sci Eng Technol 4:807–816
